# Therapeutic pressure drives the evolution of a protective ecotype characterized by AR-loss-induced senescence in prostate cancer

**DOI:** 10.7150/thno.134940

**Published:** 2026-05-11

**Authors:** Limin He, Jun Jiang, Shaojie Liu, Hongtao Song, Tong Lu, Zhihao Hu, Yu Li, Hai Zhu, Yike Zhou, Zhengxuan Li, Fa Yang, Keying Zhang, Rui Zhang, Tao Wu, Kai Gan, Bin Zhao, Jingliang Zhang, Dailing Si, Rui Zhang, Changhong Shi, Weihong Wen, Donghui Han, Chao Xu, Weijun Qin

**Affiliations:** 1 Department of Urology, Xijing Hospital, Air Force Medical University, Xi'an, China.; 2 Department of Urology, The 900th Hospital of Joint Logistic Support Force, PLA, Fuzhou, China.; 3 Department of Health Service, Base of Health Service, Air Force Medical University, Xi’an, China.; 4 Department of Urology, Institute of Surgery Research, Daping Hospital, Army Medical University, Chongqing, China.; 5 Military Medical Innovation Center, Air Force Medical University, Xi’an, China.; 6 State Key Laboratory of Holistic Integrative Management of Gastrointestinal Cancers, Department of Immunology, Air Force Medical University, Xi’an, China.; 7 Division of Cancer Biology, Laboratory Animal Center, Air Force Medical University, Xi'an, China.; 8 Institute of Medical Research, Northwestern Polytechnical University, Xi'an, China.

**Keywords:** castration-resistant prostate cancer, TME ecotype, cancer-associated fibroblasts, senescence, non-canonical NF-κB signaling

## Abstract

**Rationale:**

Prostate cancer treatment relies heavily on androgen deprivation therapy, yet the progression to a lethal treatment-resistant state presents a nearly universal clinical challenge. While tumor-intrinsic changes are well documented, the manner in which the broader tumor microenvironment dynamically reorganizes into distinct macroscopic ecological states under therapeutic pressure remains elusive. This underscores that dismantling specific therapy-induced TME niches may represent a promising strategy for CRPC treatment.

**Methods:**

We constructed a comprehensive single-cell atlas comprising 399,276 cells from 133 clinical samples to systematically investigate sample-level microenvironmental heterogeneity. Integrative bioinformatics and a meta-analysis of 1,259 patients were utilized to validate the clinical relevance. The upstream regulatory role of the androgen receptor on the NF-κB2/p52 pathway in cancer-associated fibroblasts was elucidated and validated using CRISPR-Cas9, ChIP-qPCR, and dual-luciferase reporter assays. Functional studies and therapeutic strategies targeting this axis were conducted using gain- and loss-of-function assays, and evaluated through *in vitro* organoid co-cultures and *in vivo* transgenic and xenograft mouse models.

**Results:**

We demonstrated that hormonal therapy drives a convergent systemic evolution toward a specific treatment-refractory ecological state termed Ecotype 4. Rather than isolated cellular events, this highly malignant ecosystem is characterized by a TGF-β-driven rigid vascular-stromal barrier enforcing immune exclusion and the prominent accumulation of an androgen receptor-negative senescent fibroblast population. Mechanistically, we identified that the loss of the androgen receptor releases a physiological brake on the non-canonical NF-κB pathway, forcing these fibroblasts into a pro-tumorigenic senescence phenotype. Importantly, pharmacologically blocking this NF-κB2/p52 pathway with the inhibitor SN52 reverses the supportive nature of this niche and restores sensitivity to standard antiandrogens *in vivo.*

**Conclusions:**

This study demonstrates that castration resistance is driven by the dynamic, systemic evolution of the microenvironment into a highly structured protective ecotype. Targeting the therapy-associated stromal p52 senescence switch effectively dismantles this ecological sanctuary and offers a new valued therapeutic strategy for advanced CRPC.

## Introduction

Despite the initial clinical efficacy of androgen deprivation therapy (ADT), advanced prostate cancer (PCa) almost inevitably progresses to a lethal castration-resistant prostate cancer (CRPC) 1-4. Although current therapeutic strategies primarily target tumor cell–intrinsic pathways, increasing evidence supports the “seed and soil” hypothesis, emphasizing the critical role of the tumor microenvironment (TME) in driving therapeutic resistance 5-7. Androgen deprivation not only reprograms tumor cell biology but also induces extensive remodeling of the surrounding microenvironment, reshaping stromal, vascular, and immune compartments in ways that may promote tumor persistence and progression 8-10. Nevertheless, the evolutionary trajectory of the TME under sustained ADT pressure, and the mechanisms through which these microenvironmental adaptations facilitate resistance, remain incompletely defined.

The TME is increasingly recognized as a dynamic and highly organized ecosystem in which distinct cellular populations coalesce into functional “ecotypes” that shape tumor behavior 11, 12. However, the principles governing ecosystem-level evolution of the PCa microenvironment, from hormone sensitivity to castration resistance, remain largely undefined. Although previous single-cell studies have provided valuable insights into cellular heterogeneity, they largely offer static snapshots and do not fully resolve how individual components integrate into coordinated, therapy-adaptive ecotypes under sustained treatment pressure 13-15. As a result, the key cellular drivers and interaction networks that construct the resistant TME landscape remain insufficiently characterized.

Within this ecosystem, the tumor stroma represents the dominant structural and functional compartment, serving both as a mechanical scaffold and as a signaling hub that reinforces the immunologically “cold” phenotype of prostate cancer 16-18. While we and others have demonstrated the central role of myofibroblastic cancer-associated fibroblasts (myCAFs) in extracellular matrix remodeling 10, 19, accumulating evidence suggests that the maintenance of this exclusionary state requires coordinated interactions among multiple stromal lineages 20, 21. Therefore, defining the dynamic reorganization of the stromal compartment is essential to pinpoint the specific cellular subpopulations that consolidate and sustain the castration-resistant niche.

Recent advances have begun to clarify how the stromal compartment adapts to antiandrogen stress. ADT has been shown to promote reprogramming toward a pro-tumorigenic myofibroblastic phenotype 10 and to induce resistance-associated factors such as NRG1 6. However, beyond the established framework distinguishing tumor-promoting myCAFs from tumor-suppressive iCAFs in hormone-sensitive disease 19, the precise stromal state that drives the transition to CRPC remains undefined. Although stromal senescence is a well-recognized consequence of chemotherapy and radiotherapy 22-25, whether a similar process emerges under sustained ADT has not been explored. Given that prolonged AR inhibition induces senescence in prostate cancer epithelial cells 26-28, we hypothesized that ADT may similarly impose a senescent fate on CAFs, thereby establishing a distinct resistance niche.

To systematically interrogate this evolutionary process, we constructed a comprehensive single-cell atlas of human prostate cancer spanning the continuum from hormone sensitivity to castration resistance. Our analyses reveal that therapeutic pressure drives convergence toward a stromal-dominant malignant ecotype rather than random remodeling. Within this ecosystem, we identify a previously unrecognized population of AR-negative, senescent CAFs that anchors the resistant niche. Mechanistically, we demonstrate that AR functions as a physiological restraint on non-canonical NF-κB signaling; its loss during ADT leads to sustained NF-κB2/p52 activation and the induction of a pro-tumorigenic senescence program. Importantly, pharmacological inhibition of this pathway disrupts stromal support and restores tumor sensitivity to enzalutamide. These findings define the AR–p52–senescence axis in CAFs as a therapeutically actionable vulnerability in castration-resistant prostate cancer.

## Materials and Methods

### Single-cell RNA-seq data collection, integration and preprocessing

We compiled single-cell RNA sequencing data from 133 prostate samples obtained from 103 donors across 10 publicly available datasets (Table S1). These samples were categorized into six groups: Health, Adjacent, HSPC, mHSPC, CRPC, and mCRPC. Prior to quality control, the dataset comprised a total of 548,245 cells. Cell quality was rigorously assessed based on several criteria: cells were retained if their total UMI count was below 30,000, the number of detected genes ranged between 300 and 7,000, and the percentage of mitochondrial gene counts was less than 20%. Furthermore, genes detected in fewer than 200 cells and cells with fewer than 3 detected genes were filtered out. Potential doublets were subsequently identified using the Scrublet method, assuming an expected doublet rate of 0.08. Cells predicted to have a doubletScore exceeding 0.25 were excluded from downstream analysis. The filtered gene expression matrix was normalized using the default parameters of the Seurat NormalizeData function. Then, we regressed out the effect of mitochondrial gene percentage and scaled each gene to unit variance before calculating the top 50 principal components (PCs) using the pca function. To mitigate potential batch effects introduced by integrating multiple datasets, the Harmony algorithm was applied, with ‘sample’ specified as the technical covariate for correction. After quality control, a total of 399,276 cells were retained for downstream analysis. As immune and stromal cells from different patients mixed well, we did not observe an obvious batch effect.

### Cell clustering and annotation

Clustering was performed using the FindClusters function within the Seurat R package, exploring resolutions ranging from 0.3 to 2. UMAP was employed for dimensionality reduction and visualization. The optimal resolution for identifying major cell types was determined when distinct clusters corresponding to these types clearly emerged, rather than merging at lower resolutions. Major cell types were initially annotated based on established marker genes, including markers for lymphoid lineage (CD8^+^ T, CD4^+^ T, Tregs, NK, and B cells: CD3D, CD8A, CD4, FOXP3, KLRF1, NKG7, CD79A, JCHAIN), myeloid lineage (Monocytes, Macrophages, Dendritic Cells, Mast Cells: CD14, CD16, CD68, FCN1, CD1C, LAMP3, BST2, TPSAB1, and S100A8), stromal cells (Endothelial cells and Fibroblasts: VWF, RGS5, DCN, COL1A1), and epithelial cells (EPCAM). Within the epithelial cell population, malignant cells were further distinguished from non-malignant cells by inferring large-scale copy number variations per cell using the inferCNV (v1.3.3) R package, utilizing T cells as the reference for CNV estimation.

To gain deeper insight into the heterogeneity within major cell types, a second round of clustering was performed to delineate subclusters within the TME. To avoid potential noise and dissociation-induced artifacts, a panel of 1927 genes associated with mitochondria (37 genes), heat shock proteins (117 genes), ribosomes (1526 genes), and ATP synthesis (247 genes) were excluded from this analysis (Table S2). Given the variability in cell numbers and inherent characteristics across different major cell types, distinct clustering parameters were applied. For T cell clustering, the top 20 PCs were selected from 1500 highly variable genes (HVGs) at a resolution of 1. For B cell clustering, the top 10 PCs were selected from 2000 HVGs at a resolution of 0.8. For NK cells, the top 10 PCs were selected from 1500 HVGs at a resolution of 0.6. For myeloid cells, the top 20 PCs were selected from 1500 HVGs at a resolution of 1.4. For pericytes and fibroblasts, the top 10 PCs were selected from 1500 HVGs at a resolution of 1. For endothelial cells, the top 40 PCs were selected from 2000 HVGs at a resolution of 1. After examining the top differentially expressed genes for each initial cluster, clusters exhibiting highly overlapping functionally related gene expression profiles were manually merged to define the final subclusters. Consequently, we identified a total of 4 B cell clusters, 2 plasma cell clusters, 10 CD4 T cell clusters, 10 CD8 T cell clusters, 9 NK cell clusters, 16 myeloid cell clusters, 5 CAFs, 6 pericyte clusters, 9 endothelial cell clusters, and 1 SMC cluster. Subsequently, differentially expressed genes (DEGs) were identified for each cluster using the FindMarkers or FindAllMarkers functions, employing a Wilcoxon rank-sum test. P-values were adjusted using Bonferroni correction, with a threshold of *P* < 0.05 considered significant. The top 50 most significantly expressed genes (ranked by log fold change) for each cluster were designated as cluster-specific signature gene sets for downstream analyses (Table S3).

### Bhattacharyya distance analysis

To quantitatively assess the transcriptomic differences between predefined cell populations across clinical groups, we utilized the Bhattacharyya distance metric on low-dimensional embeddings. Single-cell RNA-sequencing data were first processed and integrated using Harmony to obtain batch-corrected embeddings capturing the top 30 principal components. Only cell subclusters with more than 200 cells per group were included in the analysis to ensure robust statistical power. For each eligible cell type, we randomly subsampled equal numbers of cells from the corresponding clinical groups to mitigate sampling bias. Bhattacharyya distances were computed between the embedding distributions of these matched cell populations using the dim_dist function from the distdimscr R package, with 50 cells randomly sampled per iteration to estimate the distributional overlap in the 30-dimensional space. To control for potential stochastic effects, we performed 100 repeated measurements per cell type, including a control set wherein cells were randomly sampled to generate a null distribution of distances. The difference between observed and null Bhattacharyya distances was calculated to evaluate the significance of group-wise transcriptomic divergence. Statistical comparisons and visualization of distance differences across cell types were subsequently conducted using violin and box plots. A larger increase indicates less similarity between tissues.

### Tissue preference analysis of clusters

To assess the distribution of each major cell population and its corresponding subclusters across different groups, we calculated the ratio of observed to expected cell counts (Ro/e) for each cluster within each group. The expected cell counts were determined based on a chi-square test. A Ro/e value greater than 1 was interpreted as enrichment of the respective cluster within the specific group.

### Pathway analysis

Gene set enrichment analyses for clusters were performed by enricher function from clusterProfiler R package (v4.10.1) with parameter “qvalueCutoff = 0.05”. Gene sets were downloaded from Molecular Signatures Database (MSigDB v2024.1.Hs).

We utilized PROGENy (v1.24.0) to estimate signaling pathway activities, setting the parameter ‘top’ to 500 while keeping other settings at their defaults. The resulting PROGENy scores were averaged for cells within each cluster and subsequently visualized. To identify pathways with differential activation among subsets, we performed Wilcoxon rank-sum tests comparing each subset against the others. Pathways exhibiting an adjusted P value below 0.01 were deemed significantly dysregulated.

Pathway analysis for subclusters was conducted using the fgsea R package (v1.28.0). Key pathways were selected based on those commonly shared among the top differentially expressed genes within each subcluster. Genes were ranked according to the log2 fold change of mean expression between groups from the differential expression analysis. Pre-ranked gene set enrichment analysis was performed with 1,000 permutations. Enriched pathways were identified using the KEGG dataset from MSigDB, and pathways with an adjusted *P* value below 0.05 were considered statistically significant.

### Lineage trajectory analysis

To comprehensively reconstruct cellular differentiation trajectories, we employed three complementary computational approaches: pyVIA, Diffusion Map, and Monocle3. First, pyVIA (v0.2.4) was applied to the Harmony-corrected embeddings. A cluster graph was constructed using edge pruning and probabilistic modeling (with edgepruning_clustering_resolution_local = 1) to infer lineage relationships and pseudotime without explicitly defining a root. Trajectories were visualized using the streamplot function. Second, Diffusion Map analysis was performed using the destiny R package (v3.16.0). Harmony-corrected embeddings served as input to compute diffusion components, capturing the intrinsic geometry of the cellular manifold. The first three diffusion components were extracted to calculate diffusion pseudotime. Finally, Monocle3 (v1.3.1) was utilized to validate the trajectories. The Seurat object was converted to a cell_data_set, and the principal graph was learned on the UMAP space using learn_graph. Cells were ordered via order_cells, with the root node manually selected based on biological prior knowledge.

### TME transcriptional heterogeneity analysis

To dissect the heterogeneity of the tumor microenvironment among patients, we performed hierarchical clustering of patients using the R package “pheatmap” (v1.0.12). Euclidean distances between patients were calculated based on cell subtype abundances, followed by clustering using the ‘ward.D’ method. To validate the robustness of this clustering and to identify representative cell subtypes, we further classified patients into five groups using the R package NMF (v0.27), based on the percentage of each subcluster within all microenvironmental cells. The input matrix consisted of cell subtype proportions per sample after min-max normalization, with subtypes exhibiting zero variance across samples removed to ensure data quality. To determine the optimal factorization rank (k), we performed NMF across ranks 2 to 10, each repeated 100 times to assess stability via cophenetic correlation coefficients. Based on these metrics and biological interpretability, rank k = 4 was selected for downstream analysis. The final NMF model was run with 500 iterations using the brunet method. We extracted representative cell subtypes for each module with the extractFeatures() function and assigned samples to modules using predict().

### Cell-cell interaction analysis

Intercellular communication networks were inferred using the CellChat R package (v1.6.1). To identify potential ligand activity for inducing the expression of characteristic genes in specific cell subclusters, we utilized the NicheNet R package (v2.2.0). The characteristic genes of different cell subclusters served as the target gene list (Table S4). Potential ligands were predicted using the predict_ligand_activities function, and the top 10 ligands are depicted.

### SCENIC regulon analysis

We employed the pySCENIC Python package (v0.12.1) with default parameters to identify transcription factors characterizing each cell subcluster. Regulon Specificity Scores (RSS) were then computed using the calcRSS function available in the SCENIC R package.

### Calculation of gene signature scores based on scRNA-seq data

In addition to the previously described subcluster-specific signature gene sets, other gene sets utilized in this study were obtained from published research and downloaded from the Molecular Signatures Database (MSigDB v2024.1.Hs). Comprehensive information on these gene sets is provided in Table S5. To determine TME group-specific signature genes, we first filtered the marker genes for each cell subcluster based on a significance threshold of p_val_adj ≤ 1e-4. Genes were then ranked by avg_log2FC, and the top 20 genes from each subcluster were selected. These selected genes were subsequently integrated, and group-specific signature genes for each TME group were defined based on their expression preference across these groups.

For calculating gene set scores at the single-cell level based on the scRNA-seq data, we employed the AddModuleScore function within the Seurat R package to score individual cells, using its default parameters.

### Spatial transcriptomics (ST) analysis

Spatial transcriptomics data were obtained from GSE230282. Spatial transcriptomics data were analyzed using the Scanpy and Squidpy Python libraries. Following standard quality control, raw counts were normalized and log-transformed. Dimensionality reduction was performed using PCA and UMAP, followed by Leiden clustering. To map the spatial distribution of ecotypes identified in our scRNA-seq analysis, we calculated signature scores for each ecotype using the score_genes function based on their marker genes signatures. The resulting signature scores and the expression of individual genes were visualized on the high-resolution tissue histology images to assess their spatial heterogeneity.

### Bulk RNA-seq data analysis

Total RNA was extracted using TRIzol reagent, which was performed by LC Bioinformatics Co., Ltd. (Hangzhou, China). RNA-seq was performed on an Illumina NovaSeq 6000 system, and the resulting reads were aligned to the reference genome (GRCh38) using HISAT2. Differential expression was assessed by principal-component analysis and visualized by FPKM expression heatmap. DEGs were analyzed for gene set enrichment analysis (GSEA) with R packages: fgsea (v1.28.0) and msigdbr (v7.5.1) via the pre-ranked method with 10,000 permutations, based on the adaptive multilevel splitting Monte Carlo approach. All *P* values were adjusted with the Benjamini–Hochberg method. The list of significance was operated by setting fold changes threshold at level of 1.5 and *P* < 0.05.

Additionally, we assembled a cohort of 2661 samples from publicly available human bulk prostate cancer datasets. Bulk RNA-seq expression data for TCGA cohorts were downloaded from the National Cancer Institute (NCI) Genomic Data Commons (NCI-GDC; https://gdc.cancer.gov). Information regarding download sources, data composition, and prognostic details for other datasets is summarized in Table S1.

To validate the relative abundance of COL4A1^+^CAPs, particularly in CRPC, we utilized the previously determined gene set for this subcluster. Each sample was scored using the GSVA R package (v1.50.5). Meta-analysis with a random effects model was subsequently performed, and a forest plot was generated to summarize the findings. Furthermore, we applied the CIBERSORT method within the IOBR R package (v0.99.0) to perform deconvolution of the bulk data. To assess the correlation between the deconvoluted cell type frequencies and the relative abundance of cell populations, we employed the Spearman rank correlation coefficient, as the test data exhibited a non-normal distribution.

To infer transcription factor (TF) activities from bulk RNA-seq data, we utilized the decoupleR R package (v2.8.0). First, gene expression values (FPKM) were log2-transformed, and differential expression analysis was performed using the limma package to obtain t-statistics comparing the treatment group (FAD/Experiment) to the control group. We employed the CollecTRI resource (a comprehensive database of gene regulatory networks) as the prior knowledge network. TF activities were estimated using the Univariate Linear Model (ULM) method (run_ulm function), which infers regulator activities based on the weighted expression changes of their downstream target genes.

### Survival analysis

To evaluate the impact of the relative abundance of cell subclusters and TME groups on survival, we scored samples in the bulk dataset using the previously determined marker gene sets. Survival analysis was performed utilizing the R packages ‘survival’ (v3.6-4) and ‘survminer’ (v0.4.9). Patients from the TCGA cohort were grouped based on the module scores of these marker genes, as determined by the surv_cutpoint function. Kaplan-Meier survival curves were subsequently plotted using the ggsurvplot function. The statistical significance of the differences was assessed using a Cox proportional hazards regression model, adjusting for age and sex. *P* value of less than 0.05 was considered statistically significant.

### Human samples

All research involving human samples at Xijing Hospital, Air Force Medical University, was approved by the Ethics Committee of Xijing Hospital and performed in accordance with the 1964 Declaration of Helsinki and its later amendments or comparable ethical standards. Written informed consent was obtained from all the subjects. Sample diagnoses were validated by pathologists. Formalin-fixed paraffin-embedded sections were processed for multiplex immunofluorescence analysis.

### Mice

All mice were maintained in a specific-pathogen-free facility, and all related protocols were performed in compliance with the Guide for the Care and Use of Laboratory Animals and were approved by the Institutional Animal Care and Use Committee of Air Force Medical University. *PbsnCreERT; Pten^-/-^; Trp53^-/-^* transgenic mice and *Col1a2CreERT* mice and *Ar^-/-^* mice were obtained from Shanghai Model Organisms Center, Inc.

### Cell lines

Human PCa lines LNCaP, 22Rv1 and human embryonic kidney cell line 293T were obtained from the American Type Culture Collection (ATCC). Human primary CAFs were dissociated from tumor samples obtained from patients with prostate cancer at Xijing Hospital, Air Force Medical University. Mouse primary CAFs were dissociated from 8- to 12-week-old *PbsnCreERT; Pten^-/-^; Trp53^-/-^* mouse prostate tissues as described previously 29. The primary stromal cells were cultured in DMEM/F12 (Gibco) containing 10% FBS. All the experiments in this study used fresh primary CAFs within three passages after single-cell dissociation from prostates. For fluorescent labeling, CAFs were transduced with a lentiviral vector expressing EGFP. For full androgen deprivation (FAD) conditions, cells were cultured in phenol red-free RPMI-1640 medium supplemented with 10% charcoal-stripped serum (CSS, Gibco, USA). Cells were incubated at 37 °C in a 5% CO2 atmosphere. All cell lines were authenticated using short tandem repeat (STR) analysis, routinely checked for mycoplasma contamination and cultured less than 2 months after each thawing. Cells were treated with SN52 (#HY-P3229, MCE) following the manufacturer’s instructions.

### Lentiviral transduction and generation of stable CRISPR–Cas9 knockout and shRNA knockdown cell lines

For CRISPR–Cas9-mediated knockout of AR, sgRNAs targeting conserved exons of human *AR* and mouse *Ar* were designed using *crispor.tefor.net*. The shRNAs targeting human/mouse AR and MAP3K14 (NIK) were designed from Sigma-Aldrich recommendations. The sgRNAs and shRNAs sequences are listed in Table S6. These sgRNAs and shRNAs were subsequently cloned into the lentiCRISPRv2 vector (#52961, Addgene) or pLKO.1-TRC cloning vector, respectively. For lentiviral production, HEK293T cells were co-transfected with the lentiCRISPRv2 or pLKO.1 construct (10 μg), packaging plasmid psPAX2 (7.5 μg), and envelope plasmid pMD2.G (2.5 μg) using PEI transfection reagent. Viral supernatants were harvested at 48 and 72 h post-transfection, pooled, filtered through a 0.45-μm filter, and concentrated by ultracentrifugation (25,000 rpm, 90 min, 4 °C). The viral pellets were resuspended and stored at -80 °C. Primary CAFs were transduced with the concentrated lentivirus in the presence of 8 μg/mL polybrene (#TR-1003, Sigma). Forty-eight hours post-infection, cells were selected with 2 μg/mL puromycin (Thermo Fisher Scientific) for 5–7 days to establish stable pools. The knockout efficiency was subsequently validated by RT-qPCR.

### Cell proliferation assays

Conditioned medium was collected from control and AR-knockout CAFs cultured in FAD medium for 48 h. The supernatant was clarified by centrifugation and filtered through a 0.22-µm membrane. For the proliferation assay, 22Rv1-EGFP cells were seeded and treated with a 1:1 mixture of fresh medium and the respective CM. Cell growth was monitored over 8 days by quantifying the GFP-positive confluence using the IncuCyte S3 Live-Cell Analysis System (Sartorius). At the endpoint, cells were harvested, fixed, permeabilized, and stained with APC-conjugated Ki-67 antibody (#151231, Biolegend). The percentage of Ki-67 positive cells within the single, EGFP-positive population was analyzed using a NovoCyte flow cytometer (ACEA Biosciences) and FlowJo software.

### Isolation and culture of mouse prostate tumor organoids

The isolation and culture of mouse prostate tumor organoids was performed as described previously 30. Briefly, mouse prostate organoids were generated from *PbsnCreERT; Pten^-/-^; Trp53^-/-^* mice using a prostate cancer organoid culture kit (#abs9909, Absin) following the manufacturer’s instructions. Tumor tissues were minced and digested with collagenase at 37 °C for 30 min. The suspension was filtered through a 100-μm strainer and centrifuged at 300 × g. The resulting cell pellets were resuspended in Matrigel (#40191ES10, Yeasen) and plated as 25-μL domes in 24-well plates. Organoids were maintained in complete mouse prostate organoid medium and passaged when reaching 200–500 μm in diameter.

### Organoids and CAF co-culture

Organoids were labeled with mCherry and CAFs with EGFP via lentiviral transduction. For co-culture, EGFP-CAFs were seeded in 24-well glass-bottom plates at a density of 2 × 10⁴ cells/well. After adherence, mCherry-organoids were suspended in Matrigel (#40191ES10, Yeasen) and seeded atop the CAF layer. Co-cultures were maintained in an androgen-deprived organoid medium for 7 days. For fluorescence detection of organoids, live-cell spinning-disk confocal imaging was performed using an OLYMPUS inverted microscope (IXplore SpinSR, Japan). Organoid diameters were measured using ImageJ software.

### Tumor xenograft and bioluminescence imaging

For subcutaneous injections, 6- to 8-week-old male BALB/c nude mice were obtained from Beijing Vital River Laboratory Animal Technology Co., Ltd. 1×10^6^ 22Rv1 cells mixed with 3×10^6^ pre-induced CAFs were resuspended in 100 µL of a 1:1 solution of serum-free medium with Matrigel (#40189ES10, Yeasen) and injected subcutaneously into the flanks of male nude mice. For the orthotopic syngeneic model, *PbsnCreERT; Pten^-/-^; Trp53^-/-^* organoids were implanted into the anterior prostate lobes of* Col1a2CreERT; Ar^-/-^* or control* Ar-wt* host mice. To induce fibroblast-specific *Ar* deletion, host mice were administered tamoxifen (Sigma-Aldrich, 75 mg/kg) via intraperitoneal (i.p.) injection for 5 consecutive days, two weeks prior to tumor implantation. Surgical castration was performed to mimic ADT. After castration, mice were randomized into treatment cohorts. Enzalutamide was administered daily by oral gavage at a dose of 10 mg/kg. For combination therapy, the NF-κB2/p52 peptide inhibitor SN52 (#HY-P3229, MCE) was dissolved in PBS and administered i.p. at 1mg/kg daily. Control mice received the corresponding vehicle. Due to differential tumor growth rates, treatments were initiated at the time points indicated in the figure legends. Tumor volume (V) was calculated using the following equation: V = (length × width^2^)/2. For bioluminescence imaging, D-luciferin (75 mg/kg; #HY12591, MCE) was intraperitoneally administered and an *In Vivo* Imaging System (IVIS) (PerkinElmer) was used to acquire images of mice in the prone position 5 minutes after injection. All mice were sacrificed before tumor size reached 2 cm.

### RT-qPCR

Total RNA was extracted using TRIzol reagent (#12183555, Invitrogen) and quantified using a NanoDropTM 2000 (Thermo Fisher Scientific, USA). Subsequently, cDNA was synthesized using the cDNA Synthesis SuperMix (Yeasen) according to the manufacturer’s protocol. Quantitative real-time PCR (qPCR) was performed according to the manufacturer’s instructions, utilizing the SYBR qPCR Master Mix Kit (#11188ES03, Yeason). Data acquisition and analysis were managed using Bio-Rad CFX Manager 3.1 software. The relative mRNA expression level of the target gene was calculated using the ΔΔCT method. The primer sequences are listed in Table S6.

### Chromatin immunoprecipitation (ChIP-qPCR)

The chromatin immunoprecipitation (ChIP) assay was performed using the Chromatin Immunoprecipitation CHIP Kit (#JKR23002A, GENECREATE), according to the manufacturer’s instructions. Briefly, CAFs were cultured in phenol red-free medium containing 5% charcoal-stripped FBS and treated with 10 nM R1881 or vehicle for 4 hours to induce AR nuclear translocation. Cells were then harvested, cross-linked with 1% formaldehyde at room temperature for 10 minutes, and quenched with glycine. Cells were lysed in SDS Lysis Buffer containing protease inhibitors and DTT. Chromatin was fragmented by sonication (18% power, 1 s on/1 s off for 15 min) to generate fragments of 200–700 bp. After centrifugation, the supernatant was diluted with ChIP Dilution Buffer, and 2% was saved as the Input. The remaining chromatin was incubated with 3–5 μg of anti-AR antibody (22089-1-AP, Proteintech) or normal Rabbit IgG antibody (#3900, Cell Signaling Technology) at 4 °C. Protein A/G magnetic beads were added the following day and incubated for another 4–6 h at 4 °C. The beads were washed sequentially with Wash Buffers 1–4. Chromatin was eluted, and cross-links were reversed by incubation with NaCl and Proteinase K at 65 °C for 3 h, following RNase A treatment at 37 °C. DNA was purified using spin columns. The enrichment of AR on the *NFKB2* promoter was analyzed by qPCR (primers in Table S6) and normalized to the Input DNA.

### Dual luciferase reporter assay

Cells were seeded in 48-well plates at a density of 1×10^4^ cells per well. Upon reaching 70% confluence, cells were co-transfected using EZ Trans cell transfection reagent (Life-iLab). For each well, the transfection mixture contained 100 ng of pGL3-NFKB2 promoter reporter vector, 5 ng of pRL-TK Renilla luciferase vector, and 200 ng of pCDH-AR expression vector (or empty vector control). Six hours post-transfection, the medium was replaced. Cells were then treated with 10 nM R1881 or cultured in phenol red-free medium supplemented with 5% CSS containing 10 μM Enzalutamide to simulate FAD conditions. After 24 h of treatment, cells were lysed and luciferase activity was measured using the Dual-Luciferase Reporter Assay Kit (#E1910, Promega). The relative firefly luciferase activity was normalized to *Renilla* luciferase activity.

### Western blotting

Total protein was extracted in RIPA lysis buffer containing phosphatase inhibitors (#R0010, Solarbio) at 4 °C for 2 h to ensure complete lysis. The protein concentration was determined using a bicinchoninic acid (BCA) protein assay kit (#PC0020, Solarbio). Protein samples were heated at 80 °C for 8 min, separated on 8% to 15% SDS-PAGE gels (#PG210, Epizyme), and then transferred to PVDF membranes (Millipore). The membranes were blocked with 5% non-fat milk at room temperature for 1 hour and subsequently incubated overnight at 4 °C with the specific primary antibodies (Table S7). Following this, anti-mouse/anti-rabbit horseradish peroxidase–conjugated secondary antibodies (#A0216 and #A0208, 1:10,000, Beyotime) were applied, and bands were visualized using enhanced chemiluminescence reagents (#PE0010, Solarbio). The protein bands were recorded using a Tanon 5500 imaging system.

### SA-β-gal staining

The senescence-associated β-gal (SA-β-gal) assay was performed using the Senescence β-Galactosidase Staining Kit (#C0602, Beyotime) and Senescence-Tracker™ Green Fluorescence Staining Kit with SA-Green (#C0607, Beyotime) according to the manufacturer’s instructions. β-gal quantification was performed using three biological repeat samples. Representative images are displayed. The percentage of stained cells was measured by ImageJ software.

### ELISA

The levels of secreted IL-6 in the cell culture supernatants were measured using a human IL-6 ELISA Kit (#E-EL-H6156, Elabscience) according to the manufacturer's instructions. The optical density (OD) was measured at 450 nm using a microplate reader.

### Flow cytometry

Flow cytometry was performed to analyze cell proliferation, senescence, and apoptosis. Briefly, harvested cells were treated as follows: for proliferation, cells were fixed, permeabilized, and stained with APC-conjugated anti-Ki-67 antibody (#151231, Biolegend); for senescence, cells were incubated with Senescence-Tracker™ Green Fluorescence Staining Kit with SA-Green (#C0602, Beyotime); and for apoptosis, cells were stained using the APC Annexin V/7-AAD Kit (#BB-41034, Bestbio). All samples were acquired on an ACEA NovoCyte flow cytometer, and data were analyzed using FlowJo software.

### Histology and immunostaining

Tissues from patients, mice, and organoids were collected within 30 min after tumor resection, fixed in 4% paraformaldehyde and subsequently embedded in paraffin. Sections were cut at 4 µm thickness for all histological and immunostaining procedures. For routine histological examination, sections were deparaffinized and rehydrated. Hematoxylin and Eosin (H&E) staining was performed by immersing slides in hematoxylin for 30 seconds and eosin for 1 minute. Masson's Trichrome staining was carried out according to the manufacturer's instructions (Servicebio). IHC was performed according to the manufacturer’s protocol. mIF staining was performed as previously described 31, utilizing primary antibodies also listed in Table S7. All stained slides were visualized and images were captured using the 3DHISTCH software. For fluorescent images (from mIF), quantification of signal intensity was performed using ImageJ software, ensuring consistent image settings were applied across all samples.

### Statistical analysis

All statistical analyses were performed using R (v.4.3.2), Python (v.3.10.14), or GraphPad Prism (v.9.0). For bioinformatics data, which typically do not follow a normal distribution, non-parametric tests were employed: the Wilcoxon rank-sum test for two groups and the Kruskal-Wallis test for multiple groups. For experimental data, data normality was assessed using the Shapiro-Wilk test. Parametric tests were used for normally distributed data: unpaired Student’s t-test for two-group comparisons, one-way ANOVA followed by Tukey’s post hoc test for multiple groups, and two-way ANOVA followed by Bonferroni’s post hoc test for time-course or multi-factor experiments. Categorical variables were analyzed using the χ² test or Fisher’s exact test. All statistical tests were two-sided, and *P* < 0.05 was considered statistically significant. Data are presented as mean ± SEM unless otherwise stated. No statistical methods were used to predetermine sample sizes, but sample sizes were chosen based on standard practices in the field.

## Results

### The single-cell landscape of the microenvironment during prostate cancer progression

**Figure 1:**
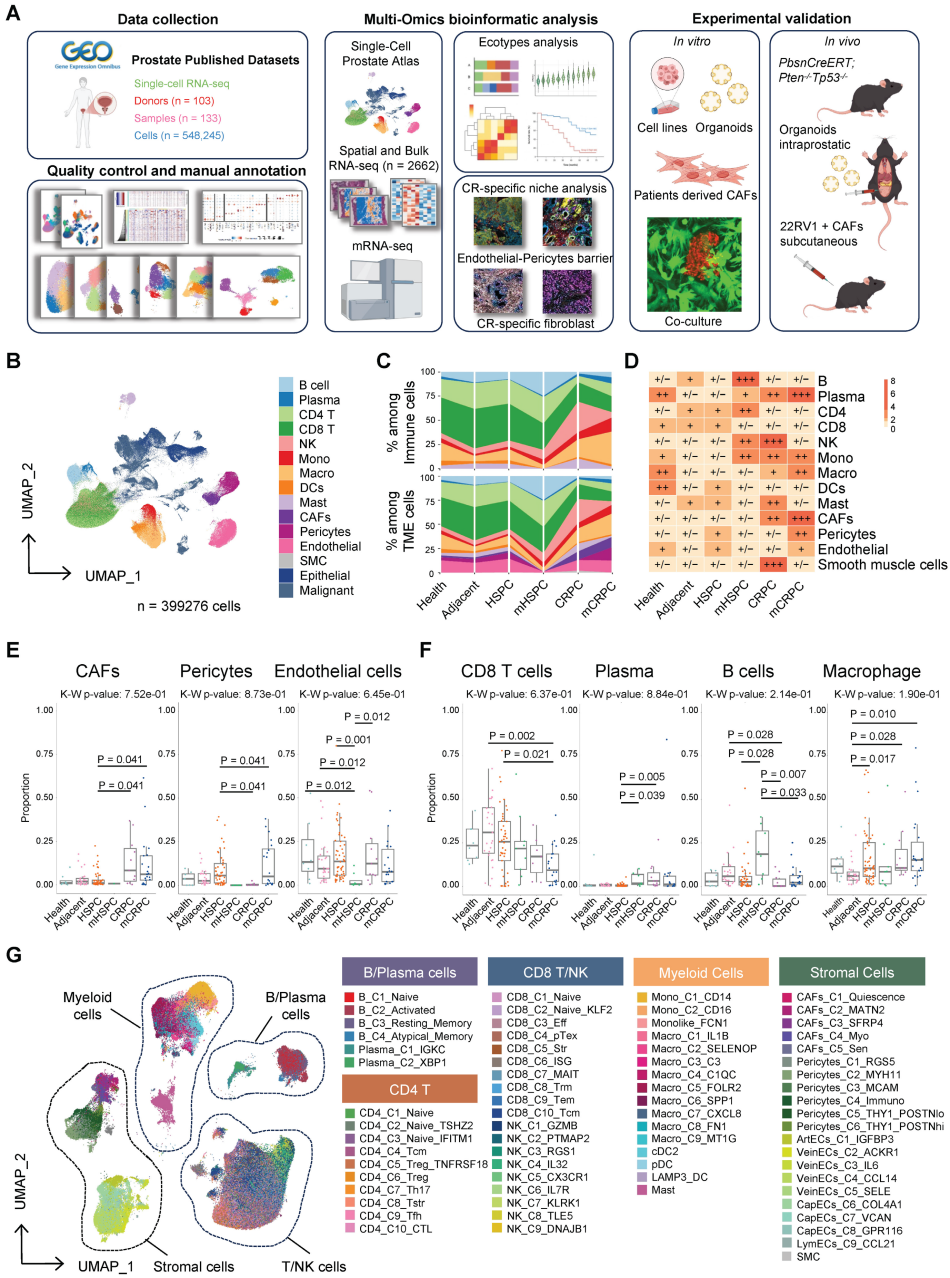
** The single-cell landscape of the microenvironment during prostate cancer progression.** (A) Schematic representation of the research framework. (B) Uniform manifold approximation and projection (UMAP) visualization of major cell types. (C) Cell type compositions of total tumor microenvironment (TME) cells (bottom) and immune cell subsets (top) across clinical tissue groups. (D) Heatmap displaying the prevalence of major cell types in each tissue, quantified by the ratio of observed to expected (Ro/e) cell counts. (E) Proportions of stromal cell lineages across tissue groups. (F) Proportions of representative immune cell subtypes across tissue groups. Overall statistical significance was determined by the Kruskal-Wallis test, with pairwise comparisons using the Wilcoxon rank-sum test; *P* < 0.05 was considered statistically significant. (G) UMAP plot delineating the identified subclusters within the integrated TME cell population.

To comprehensively delineate the heterogeneity and spatiotemporal evolution of the TME across PCa progression, we integrated published scRNA-seq datasets encompassing multiple clinicopathological states, including healthy controls (health), adjacent normal tissues (adjacent), hormone-sensitive prostate cancer (HSPC), metastatic HSPC (mHSPC), CRPC, and metastatic CRPC (mCRPC). This integrative analysis generated a single-cell atlas comprising 548,245 cells from 133 samples across 103 donors and 10 independent datasets (Figure 1A, Table S1). After rigorous quality control, 399,276 high-quality cells were retained for downstream analyses. Using canonical marker genes, cells were annotated into major lineages, including T/NK cells, B/plasma cells, myeloid cells, and stromal cells (Figure 1B and Figure S1A). Malignant epithelial cells were distinguished from benign epithelial cells through inferred copy number variation analysis (Figure S1B). Comprehensive quality assessments, including down-sampling, batch effect correction, and principal component analysis, confirmed the robustness of data integration and demonstrated that clustering patterns were driven predominantly by biological variables, such as cell identity and pathological state, rather than technical artifacts (Figure S2A–E).

To characterize dynamic TME remodeling during disease progression, we compared lineage composition across pathological stages. This analysis revealed pronounced and coordinated shifts in cellular architecture (Figure 1C–F, Figure S2F). Within the immune compartment, CD8^+^ T cells progressively declined with advancing disease, whereas myeloid populations, particularly macrophages, expanded substantially in metastatic and castration-resistant settings, consistent with prior reports of immunosuppressive reprogramming 32. Plasma cells exhibited a distinct peak in mCRPC, potentially reflecting compensatory humoral responses or tertiary lymphoid structures (TLS) formation 33. Specifically, stromal remodeling was especially prominent: CAFs and pericytes were markedly enriched in CRPC and mCRPC (Figure 1C–F). When regrouped into normal, hormone-sensitive, and castration-resistant (CR) states, Ratio of observed to expected (Ro/e) analysis confirmed significant expansion of stromal lineages, particularly CAFs and smooth muscle cells, in the CR group, accompanied by macrophage accumulation and progressive CD8^+^ T-cell depletion (Figure S3A–B). These findings define a stroma-dominant, immunosuppressed ecosystem as a hallmark of the castration-resistant niche.

To further resolve the cellular states underpinning this evolution, we performed high-resolution subclustering within each major lineage, identifying 76 distinct subpopulations, including 10 CD4^+^ T-cell, 10 CD8^+^ T-cell, 16 myeloid, and 5 CAF subsets (Figure 1G, Figure S4). This refined atlas provides the foundation for subsequent functional dissection of lineage-specific contributions to TME remodeling.

### Castration resistance in prostate cancer is accompanied by profound TME remodeling

**Figure 2:**
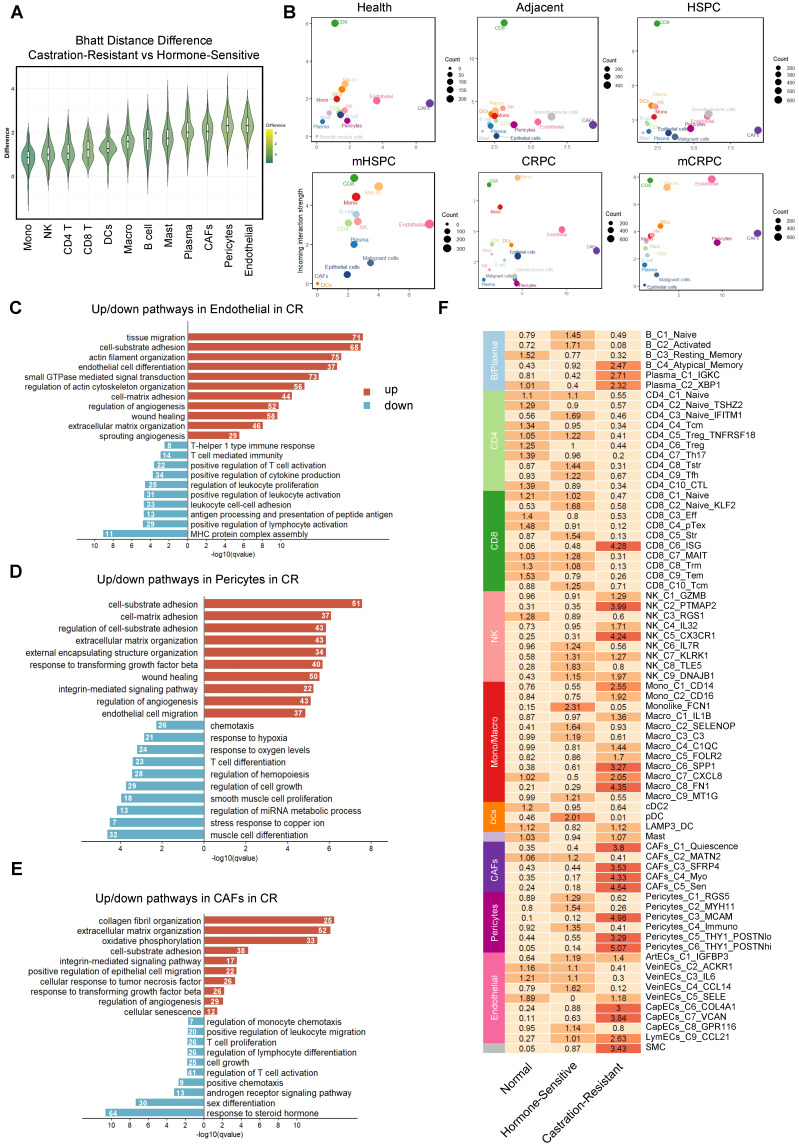
** Castration resistance in prostate cancer is accompanied by profound microenvironmental remodeling.** (A) Box and violin plots illustrating the variance in Bhattacharyya (Bhatt) distance between castration-resistant and hormone-sensitive groups across cell types (n = 100 for each cell type). (B) CellChat analysis depicting alterations in intercellular communication patterns between groups; the y-axis represents received signal strength and the x-axis indicates sent signal strength. (C-E) Bar plots showing top upregulated and downregulated pathways in stromal cell populations in the castration-resistant group compared to the hormone-sensitive group. Numbers indicate gene counts matched to corresponding biological pathways. (F) Heatmap illustrating tissue enrichment for each cell subcluster, as determined by the Ro/e score.

To identify the lineages undergoing phenotypic evolution during the transition to CR, we quantified global transcriptional divergence across major cell types using Bhattacharyya distance analysis. This unbiased approach revealed that the stromal compartment, particularly endothelial cells, pericytes, and CAFs, exhibited the greatest transcriptional shifts, far exceeding those observed in immune lineages (Figure 2A). Consistent with this finding, cell–cell communication analysis demonstrated a marked increase in both signaling input and output among stromal populations in CRPC, indicating enhanced functional activity and network centrality within the TME (Figure 2B). These results position the stroma as a dominant regulatory hub in the hormone-resistant microenvironment of PCa.

To define the functional consequences of stromal reprogramming, we performed GSEA across major lineages. Endothelial cells displayed suppression of immune surveillance programs, including MHC class I assembly and lymphocyte adhesion, alongside activation of angiogenic and invasive pathways (Figure 2C), consistent with the formation of an immune-restrictive vascular barrier. Concurrently, CAFs and pericytes converged on a shared pro-fibrotic transcriptional program marked by enrichment of ECM organization, integrin signaling, and TGF-β pathway activation (Figure 2D-E). Notably, CR-associated CAFs exhibited pronounced downregulation of AR signaling accompanied by strong senescence signatures. These coordinated alterations establish a multilayered, pro-tumorigenic ecosystem that sustains tumor growth while reinforcing immune exclusion.

To delineate the cellular subsets orchestrating TME remodeling during disease evolution, we examined the compositional architecture of the CR niche using Ro/e analysis. This revealed coordinated expansion of structural lineages, including CAFs, endothelial cells, and pericytes, together with pro-fibrotic myeloid populations, particularly SPP1^+^ and FN1^+^ tumor-associated macrophages (Figure 2F). These findings firmly establish high-grade stromal remodeling as a defining feature of the CR microenvironment. In contrast, the adaptive immune compartment exhibited a complex pattern of numerical fluctuation coupled with functional impairment. Although certain subsets, including B cells, NK cells, and ISG^+^ CD8^+^ T cells, were enriched in CR, functional analyses indicated limited anti-tumor activity. Multiple T-cell populations displayed broad transcriptional features of exhaustion (Figure S5A–B). Regulatory T cells (Tregs), particularly the CD4_C5_Treg_TNFRSF18 subset expressing TNFRSF family members such as TNFRSF18 and TNFRSF4, were enriched in the HS stage, suggesting that immunosuppressive conditioning begins early in disease progression (Figure S5C). The B-cell compartment shifted toward atypical memory and plasma cell phenotypes, consistent with attenuation of effective humoral responses (Figure 2F, Figure S5D). Similarly, the expanded NK_C9_DNAJB1 subset preferentially expressed stress-response genes and the exhaustion mediator *NR4A1* rather than canonical cytotoxic markers 34-36 (Figure S6A–D), indicating functional compromise. These coordinated alterations reinforce a stromal-dominant, immune-excluded ecosystem that physically and functionally restricts effective anti-tumor immunity during PCa progression.

### Identification of distinct TME ecotypes and characterization of a CRPC-specific stromal niche

**Figure 3:**
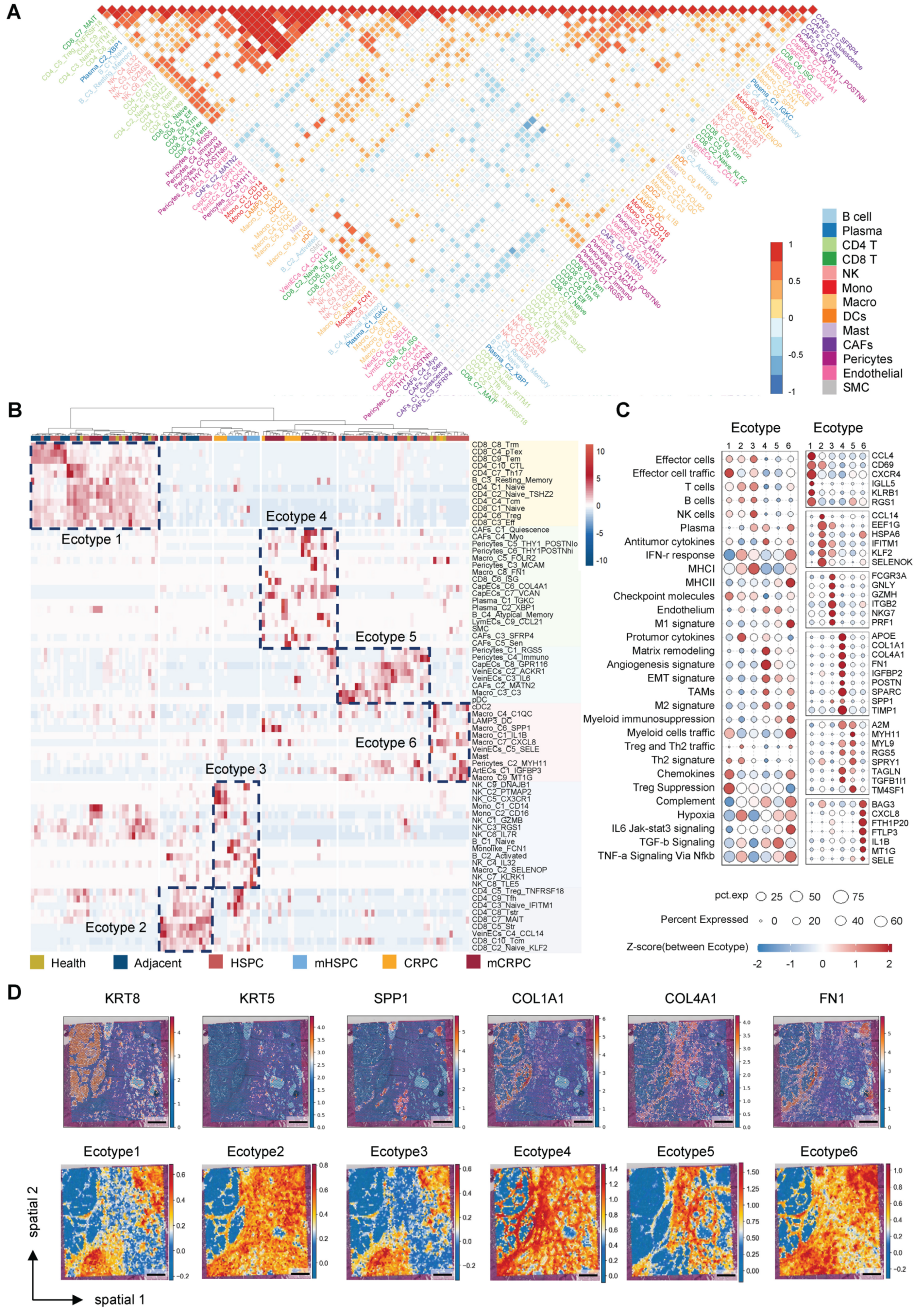
** Identification of distinct TME ecotypes and characterization of a CRPC-specific stromal niche.** (A) Correlation among 72 TME cell subclusters based on relative population abundance. P-values were computed using the Spearman correlation test with Benjamini-Hochberg correction; only correlations with *P* ≤ 0.05 are displayed, with larger or darker squares indicating stronger correlations. (B) Heatmap illustrating unsupervised patient clustering based on the relative abundance of cell subpopulations. (C) Dot plot depicting TME-associated signature scores and the expression of ecotype-specific marker genes. (D) Spatial mapping of marker genes and ecotype scores on spatial transcriptomics slides. (scale bar = 1 mm).

To elucidate the higher-order organizational principles of the PCa TME, we moved beyond simple cell-type proportions and inferred cellular communities based on co-occurrence patterns. Pairwise correlation analysis across all identified subpopulations demonstrated that the TME is not a stochastic assemblage of cells but instead segregates into distinct, interacting modules (Figure 3A). The correlation heatmap revealed a pronounced dichotomy: one module was dominated by adaptive immune populations, including CD8^+^ T cells, CD4^+^ T cells, and B cells, with strong internal positive correlations, whereas a second module consisted primarily of structural stromal populations and pro-tumorigenic myeloid subsets, such as SPP1^+^ macrophages. Importantly, these modules were mutually exclusive, as samples enriched for the stromal–myeloid complex were consistently depleted of adaptive immune effectors (Figure 3A, Figure S7A). This compositional antagonism provides direct evidence that the TME organizes into recurrent, functionally opposing ecosystems, laying the foundation for defining discrete microenvironmental ecotypes.

Unsupervised clustering of the cellular composition matrix across all samples identified six TME ecotypes (Figure 3B), each defined by a characteristic constellation of coexisting cell states that collectively trace disease progression (Figure S7B). Ecotypes 1, 2, 5, and 6 were predominantly observed in healthy, adjacent, and HSPC tissues, reflecting early-stage heterogeneity. Ecotype 1 was marked by T-cell infiltration with early exhaustion features (Figure 3C), whereas Ecotype 5 represented a relatively homeostatic stromal configuration. A transitional Ecotype 3 emerged in mHSPC, characterized by immune perturbation involving NK cells and monocytes. Strikingly, advanced CRPC and mCRPC samples converged almost exclusively toward Ecotype 4 (Figure S7B), suggesting that sustained androgen deprivation imposes strong selective pressure that funnels the TME into a uniform, therapy-refractory state. Molecular profiling defined Ecotype 4 as a highly integrated pro-tumorigenic niche, exhibiting elevated EMT, hypoxia, and TGF-β signaling scores and overexpression of stromal and immunosuppressive mediators, including SPP1 and POSTN (Figure 3C). Independent validation using NMF identified a functional module (Module 5) enriched almost exclusively in late-stage CRPC/mCRPC and driven by the same stromal populations, including CapECs_C6_COL4A1, that characterize Ecotype 4 (Figure S7C). These analyses confirm that coordinated stromal remodeling is a defining hallmark of the CR state.

To define the physical architecture underlying these computationally defined ecotypes, we projected the molecular drivers of Ecotype 4 onto spatial transcriptomics datasets (GSE230282) (Figure 3D). Spatial analysis demonstrated that key pro-fibrotic genes, including *COL1A1, COL4A1*, and *FN1*, together with the immunosuppressive mediator *SPP1*, display coordinated localization patterns and coalesce into dense fibrotic structures that encapsulate tumor clusters. This spatial organization supports the concept of a structurally reinforced niche rather than diffuse stromal activation. We next assessed the clinical relevance of this microenvironmental configuration in the independent TCGA cohort. Ecotype 4 scores increased significantly with higher Gleason scores (GS) (Figure S7D–F) and were associated with reduced progression-free survival (Figure S7G). These findings establish Ecotype 4 as both a spatially organized and clinically adverse ecosystem that actively contributes to malignant progression.

### COL4A1^+^ endothelial cells and activated pericytes cooperatively construct a vascular stromal barrier in CRPC

**Figure 4:**
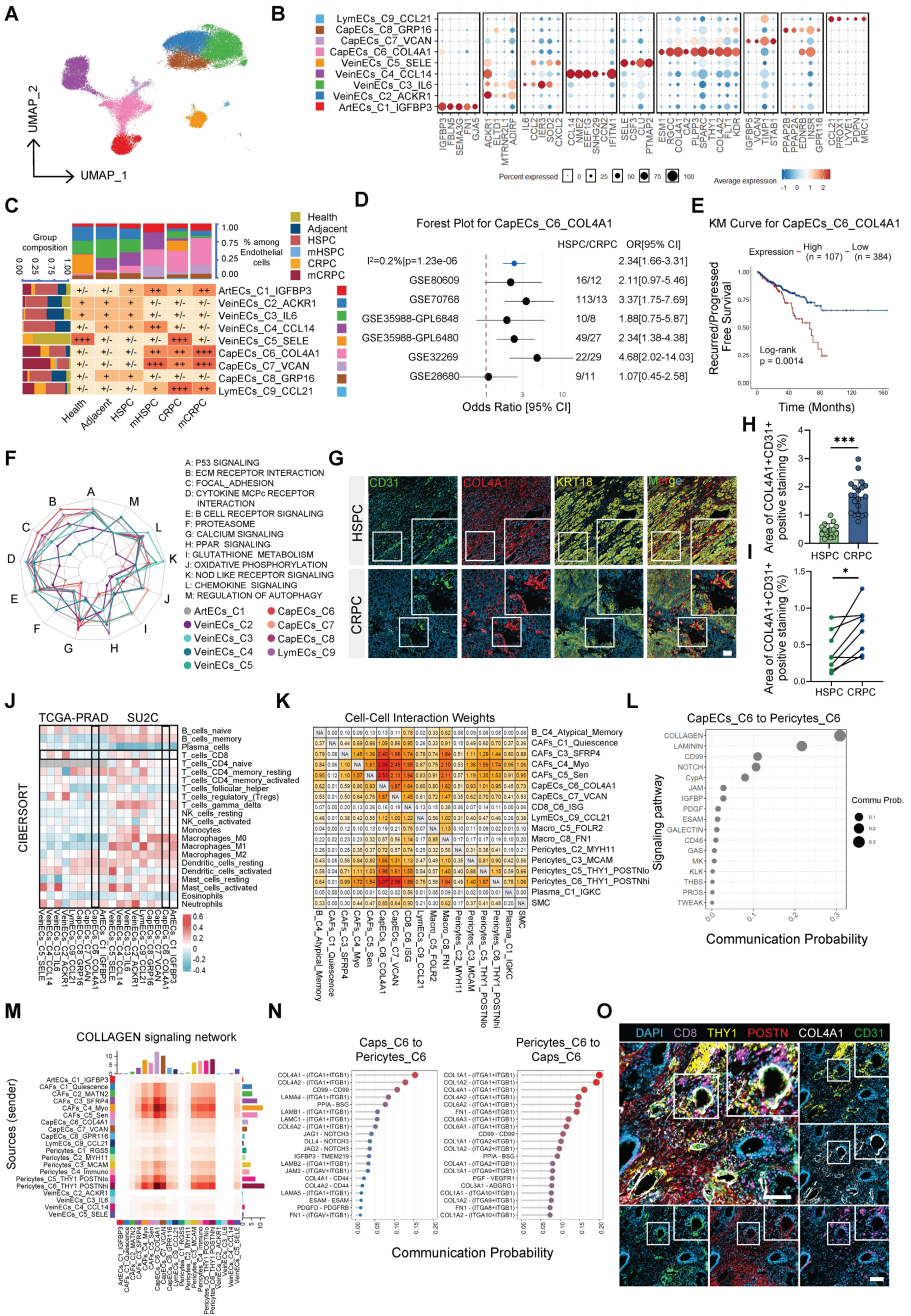
***COL4A1*^+^ endothelial cells and activated pericytes cooperatively construct a vascular stromal barrier in CRPC.** (A) UMAP visualization of endothelial cell subclusters. (B) Dot plot illustrating marker gene expression across endothelial subclusters. (C) Heatmap displaying tissue enrichment for each endothelial subcluster quantified by Ro/e score; top bars show cell compositions and left bars show tissue compositions. (D) Meta-analysis of CapECs_C6_COL4A1 scores across six datasets based on the odds ratio (OR) of CRPC versus HSPC. (E) Kaplan-Meier survival analysis of CapECs_C6_COL4A1 scores in the TCGA dataset. (F) Radar chart showcasing KEGG pathway enrichment for each endothelial subcluster. (G) Representative multiplex immunofluorescence (mIF) images of HSPC and CRPC samples showing CD31 (green), COL4A1 (red), and KRT18 (yellow); white boxes indicate regions of interest (scale bar = 100 µm). (H) Quantitative analysis of COL4A1^+^CD31^+^ area in HSPC (n=15) and CRPC (n=20) samples. (I) Quantitative analysis of COL4A1^+^CD31^+^ area in paired HSPC and CRPC samples (n=8). Statistical significance: *P < 0.05, ***P < 0.001. (J) Heatmap illustrating Spearman correlations between deconvolution scores and endothelial enrichment scores in TCGA and SU2C datasets. (K) Heatmap showing Cell-Cell Interaction Weights between stromal populations determined by CellChat. (L) Dot plot displaying top pathways from CapECs_C6_COL4A1 targeting Pericytes_C6_THY1^+^POSTN^hi^. (M) Heatmap of the COLLAGEN signaling network among stromal populations. (N) Chord diagrams illustrating molecular interactions mediating communication between CapECs_C6_COL4A1 and Pericytes_C6_THY1^+^POSTN^hi^. (O) Representative mIF images demonstrating spatial distribution of COL4A1, CD31, POSTN, THY1, and CD8 (scale bar = 100 µm).

Having identified Ecotype 4 as the convergent malignant niche, we next dissected its cellular architecture. Given the prominent contribution of endothelial cells to this ecotype (Figure 2A, Figure S7C), we performed subclustering of the endothelial lineage (Figure 4A–B). This analysis identified a distinct subcluster, CapECs_C6_COL4A1, which was markedly expanded in CRPC and mCRPC (Figure 4C, Figure S8A). This enrichment was validated in a meta-analysis of 319 patients (219 HSPC and 100 CRPC) (Figure 4D). Across independent datasets, including the TCGA cohort and a pooled analysis of 1,259 patients, elevated CapECs_C6_COL4A1 expression consistently predicted earlier recurrence and reduced progression-free survival (Figure 4E, Figure S8B).

Trajectory analysis consistently positioned CapECs_C6_COL4A1 at the terminus of the endothelial differentiation continuum (Figure S8C–D), indicating that this population represents an evolutionary endpoint associated with disease progression. Functionally, CapECs_C6_COL4A1 displayed a pronounced matrix-remodeling phenotype, with significant enrichment of ECM–receptor interaction and focal adhesion pathways (Figure 4F). To define upstream regulatory mechanisms, we integrated pathway activity and ligand–target analyses. PROGENy scoring revealed selective hyperactivation of the TGF-β signaling axis (Figure S8E), while NicheNet identified *TGFB1*, second only to *VEGFA*, as a key upstream ligand predicted to govern the transcriptional program of this subset (Figure S8F). Multiplex immunofluorescence (mIF) confirmed a substantial expansion of CD31^+^COL4A1^+^ endothelial cells in CRPC relative to HSPC tissues (Figure 4G–H). Importantly, analysis of longitudinally paired clinical samples demonstrated that this collagen-enriched vascular phenotype was specifically acquired during the transition to resistance (Figure 4I). These findings define a distinct vascular remodeling program in CRPC characterized by the emergence of a TGF-β–responsive, COL4A1^+^ capillary subset that is associated with adverse clinical outcomes.

To further clarify how CapECs_C6_COL4A1 remodels the niche, we performed deconvolution analyses in the TCGA and SU2C cohorts. CapECs_C6_COL4A1 abundance was inversely correlated with CD8^+^ T-cell infiltration and positively correlated with M2-like macrophages (Figure 4J), implicating this subset in immune exclusion during CRPC. Network analysis identified CapECs_C6_COL4A1 as a central hub within the TME, exhibiting strong bidirectional interactions with CAFs and pericytes (Figure S8G–H). Importantly, the most prominent signaling input originated from Pericytes_C6_THY1_POSTN^hi^ (Figure 4K), suggesting a coordinated endothelial–pericyte alliance.

Motivated by this observation, we performed in-depth characterization of the pericyte lineage. Pericytes_C6_THY1_POSTN^hi^ occupied a terminal differentiation state (Figure S9A–D), were significantly enriched in CRPC, and demonstrated robust activation of ECM remodeling pathways (Figure S9E–F). Survival analyses further supported their clinical relevance (Figure S9G). To identify upstream regulators, we interrogated their signaling landscape and found that, analogous to the TGFB1-driven program in endothelial cells, NicheNet analysis identified *TGFB2* as the principal ligand predicted to shape the transcriptional identity of Pericytes_C6_THY1_POSTN^hi^ (Figure S9H). These findings indicate that the TGF-β superfamily coordinates remodeling across both endothelial and perivascular compartments. Ligand–receptor analysis further revealed collagen signaling as the dominant molecular axis linking these populations (Figure 4L–N), supporting a model in which endothelial and pericyte subsets cooperatively establish a collagen-enriched physical barrier.

Finally, mIF spatial imaging demonstrated that COL4A1^+^ ECs were tightly ensheathed by THY1^+^POSTN^hi^ pericytes, forming a compact vascular–stromal structure within CRPC tissues (Figure 4O). Importantly, this configuration functioned as a spatial barrier, as CD8^+^ T cells were largely excluded from the vicinity of this complex (Figure 4O). Integrating these multidimensional data, we propose a vascular–stromal barrier model of immune evasion in CRPC. Centered on TGF-β–activated CapECs_C6_COL4A1 and reinforced by Pericytes_C6_THY1_POSTN^hi^, this barrier shapes an immunosuppressive microenvironment through coordinated collagen deposition and structural cross-linking, thereby physically restricting infiltration of cytotoxic T cells.

### Loss of androgen receptor in CAFs drives therapy resistance and tumor growth in CRPC

**Figure 5:**
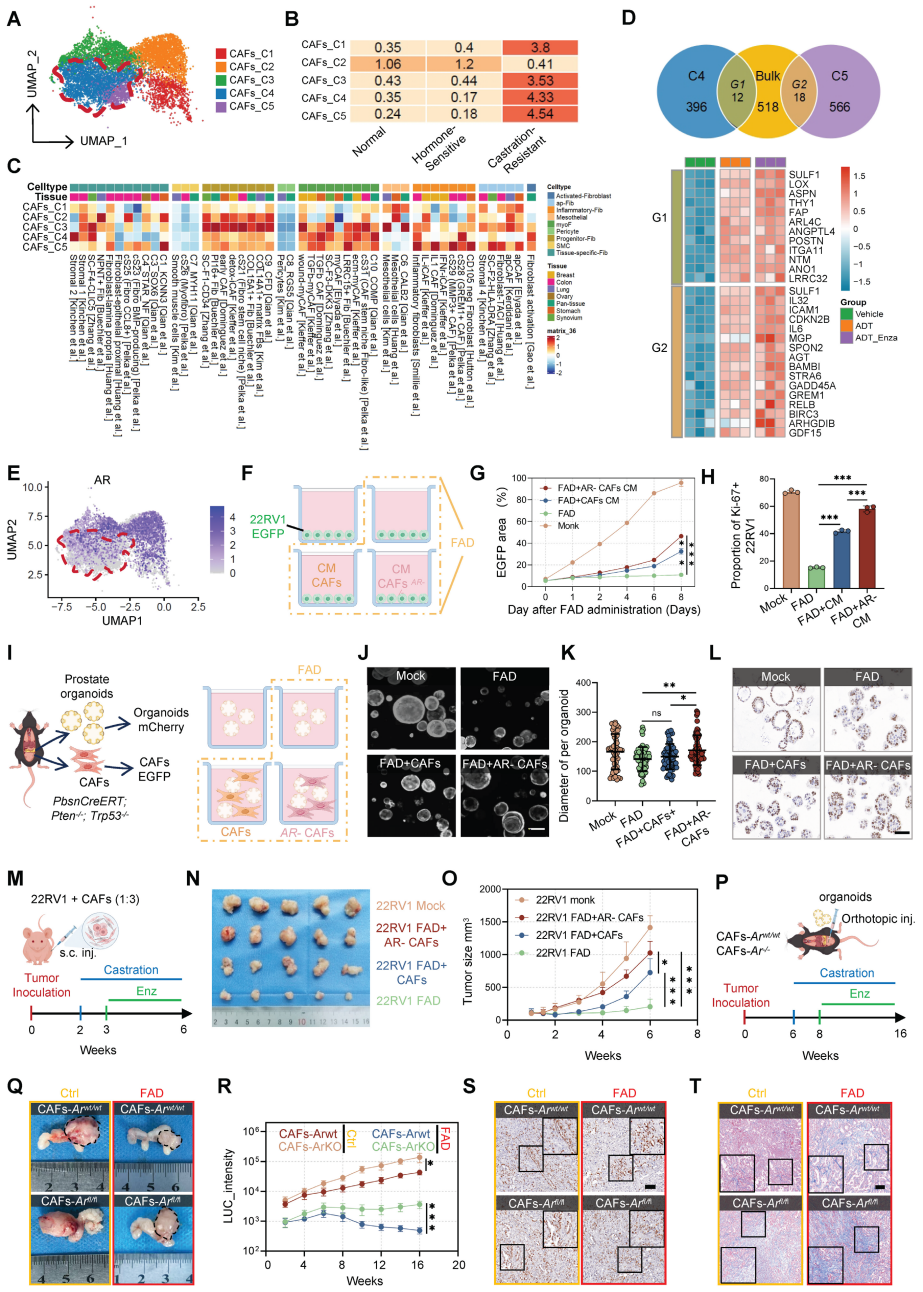
** Loss of androgen receptor in CAFs drives therapy resistance and tumor growth in CRPC.** (A-B) UMAP visualization (A) and Ro/e analysis (B) showing enrichment of clusters C4 and C5 in the hormone-resistant stage. (C) Heatmap displaying functional pathway scores of CAF subclusters. (D) Venn diagrams showing intersection between genes upregulated in C4/C5 and FAD-induced DEGs. (E) Feature plot showing AR expression in C4 and C5 clusters. (F-H) *In vitro* assessment of CAF-mediated support; schematic of co-culture (F), quantification of tumor growth by EGFP area (G), and Ki-67^+^ fraction (H). (I-L) *Ex vivo* organoid co-culture; schematic of the organoid co-culture system (I), representative images (J; scale bar = 50 µm), diameter quantification (K), and representative Ki-67 staining of organoids (L; scale bar = 100 µm). (M-O) Subcutaneous xenograft model (n=5); schematic of the 22Rv1/CAF co-injection model (M), gross images (N), and growth curves (O). (P-T) Orthotopic syngeneic model (n=5); schematic of organoid implantation (P), tumor photographs (Q), luciferase intensity (R), Ki-67 IHC (S; scale bar = 100 µm), and Masson staining (T; scale bar = 100 µm). Data are presented as mean ± SEM; statistical significance determined by one-way or two-way ANOVA. *P < 0.05, **P < 0.01, ***P < 0.001.

CAFs represent the predominant stromal population within the TME and have been strongly implicated in therapeutic resistance and metastatic progression. Nevertheless, their functional heterogeneity during the transition to CRPC remains incompletely defined. Re-clustering of our scRNA-seq dataset resolved CAFs into five discrete subpopulations, among which clusters C4 and C5 were selectively enriched in CRPC and mCRPC samples (Figure 5A–C, Figure S10A), suggesting a specific association with hormone resistance. Clinically, elevated transcriptional signatures of these subsets correlated with recurrence in patients receiving hormonal therapy (Figure S10B–C). Trajectory analysis further delineated a differentiation continuum from the secretory C5 state toward the matrix-producing C4 phenotype (Figure S10D–E), indicating that selective targeting of the matrix-remodeling C4 population alone may be insufficient to disrupt stromal support.

To determine whether androgen deprivation directly induces this phenotypic shift, we performed bulk mRNA-seq on patient-derived CAFs cultured under full androgen deprivation (FAD) conditions (CSS plus enzalutamide) (Figure S10F–G). Strikingly, the transcriptional program elicited by FAD recapitulated the gene signatures of both C4 and C5 subsets, including upregulation of matrix regulators such as *SULF1*, *LOX*, and *POSTN*, as well as secretory mediators including *IL-6*, *GDF15*, *GREM1*, and *ICAM1* (Figure 5D). These findings indicate that repression of androgen signaling drives the emergence of CRPC-associated CAF states. Consistently, AR mRNA expression was markedly reduced in CRPC-enriched subsets, particularly in C5, which was nearly devoid of AR expression (Figure 5E). This inverse relationship supports a model in which AR loss serves as a key initiating event in stromal activation during castration resistance.

To define the functional consequences of AR-deficient remodeling, we generated AR-knockout (AR-KO) primary CAFs using CRISPR–Cas9. *In vitro*, conditioned medium (CM) derived from AR-KO CAFs significantly enhanced proliferation and viability of 22Rv1 tumor cells under FAD conditions compared with medium from WT CAFs, as reflected by increased tumor area and a higher Ki-67^+^ fraction (Figure 5F–H; Figure S10H). We next isolated organoids and murine CAFs (mCAFs) from *PbsnCreERT; Pten^-/-^; Trp53^-/-^* (*Ppt*) mice. In co-culture assays, WT CAFs conferred only modest protection under FAD, whereas AR-deficient CAFs markedly rescued organoid growth and sustained proliferative activity (Figure 5I–L; Figure S10I), demonstrating that stromal AR loss functionally contributes to therapeutic resistance. To validate these findings *in vivo*, we employed a subcutaneous xenograft model subjected to FAD treatment. Tumors co-injected with AR-deficient primary CAFs exhibited accelerated growth and diminished responsiveness to therapy compared with controls (Figure 5M–O; Figure S10J), accompanied by pronounced collagen deposition (Figure S10K). To assess the impact of endogenous stromal AR deletion, we orthotopically transplanted Ppt organoids into fibroblast-specific Ar-knockout mice (*CAFs-Ar^-/-^*) (Figure 5P). Selective *AR* ablation in CAFs significantly increased tumor burden and proliferation under FAD (Figure 5Q–S; Figure S10L–M) and promoted extensive fibrotic remodeling (Figure 5T; Figure S10N). Collectively, these data establish suppression of AR signaling as a central driver of CAF phenotypic reprogramming, thereby fostering a castration-resistant TME.

### AR loss orchestrates a pro-tumorigenic senescence program via the NF-κB2/p52 axis

**Figure 6:**
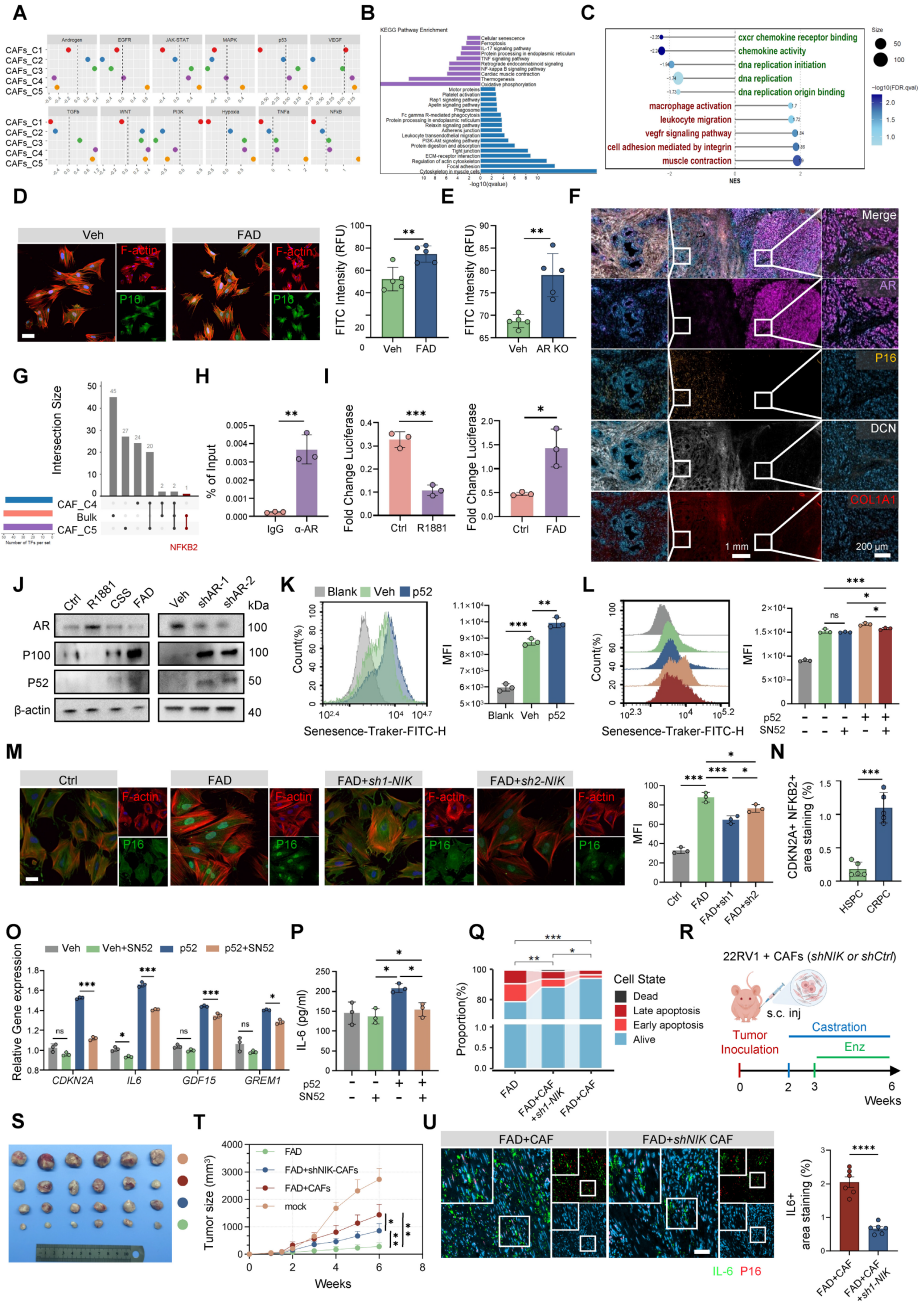
** AR loss orchestrates a pro-tumorigenic senescence program via the NF-κB2/p52 axis.** (A-C) Transcriptional profiling of CAF subclusters including a heatmap of PROGENy pathway activity scores (A), KEGG enrichment analysis (B), and functional annotation of genes in G1/G2 intersections (C). (D-E) Representative image of IF staining for p16 in CAFs upon FAD treatment (scale bar = 50 µm). (D) or AR knockout (E). (F) Representative image of mIF images showing AR/p16/DCN/COL1A1 localization in CRPC tissues. (scale bar = 1mm and 200 µm). (G) Upset plot showing a C5-specific transcription factor. (H) ChIP-qPCR analysis of AR at the *NFKB2* promoter. (I-J) Luciferase reporter assay of the *NFKB2* promoter (I) and western blot analysis of p52 expression (J) under AR-modulated conditions. (K-L) Flow cytometry analysis and quantification of cellular senescence in CAFs with p52 overexpression (K) and subsequent treatment with the p52 inhibitor SN52 (L). (M) IF staining and quantification of p16 in FAD-treated CAFs with or without *NIK* knockdown. (scale bar = 50 µm). (N) Quantification of CDKN2A^+^NF-κB2^+^ area staining in HSPC and CRPC samples. (O-P) qRT-PCR (O) and ELISA (P) analyses of SASP factor expression in p52-overexpressing CAFs treated with or without SN52. (Q) Analysis of tumor cell apoptosis following co-culture with the indicated CAFs. (R-T) Schematic of the xenograft model (R), tumor photographs (S), and growth curves (T) for mice co-injected with 22Rv1 tumor cells and control or shNIK-CAFs under FAD conditions. (n=6) (U) Representative IF images and quantification of IL6 and p16 in tumor sections from the xenograft models. (scale bar = 50 µm). Data are presented as mean ± SEM. Statistical significance was determined using one-way ANOVA or two-way ANOVA followed by post hoc tests, or unpaired t-test for two-group comparisons. *P < 0.05, **P < 0.01, ***P < 0.001.

To define the functional identity of the CRPC-specific stromal niche, we interrogated signaling dynamics across the identified CAF subsets. PROGENy analysis revealed distinct pathway activation patterns in C4 and C5, with C5 showing marked enrichment of secretory programs, accompanied by pronounced suppression of AR signaling in both subsets (Figure 6A). Intriguingly, the C5 subset also exhibited significant upregulation of p53 signaling. Given the well-established association between p53 activation and cellular senescence 37, we hypothesized that AR deprivation promotes a senescent transition in CAFs. Consistently, GSEA demonstrated significant enrichment of cell senescence signatures in C5 (Figure 6B; Figure S11A), a result further supported by bulk mRNA-seq of FAD-treated CAFs, which displayed broad activation of inflammatory and senescence-related pathways (Figure 6C).

Analysis of an independent murine dataset suggested that the C5-associated phenotype, termed SenCAFs, represents a major stromal effector of castration resistance (Figure S11B–D). To establish causality, we examined patient-derived primary CAFs and found that *AR* knockout induced a more robust upregulation of senescence-associated genes than FAD treatment alone (Figure 6D–E; Figure S11E–H), indicating that AR loss, rather than ligand deprivation per se, is the principal driver of this program. Spatial mIF analysis in CRPC patient samples and *Ppt* murine models revealed a striking inverse relationship between stromal AR expression and senescence markers: regions lacking AR were characterized by increased p16 expression, expansion of CAFs, and elevated *Il6* levels (Figure 6F; Figure S11I–J). Consistent findings in *CAFs-Ar^-/-^* mice provided *in vivo* evidence that AR loss directly precipitates stromal senescence (Figure S11K).

To identify the key transcriptional driver of this state, we intersected transcription factor activity predictions from single-cell data (using SCENIC) with bulk-level inferences (using decoupleR). This integrated analysis identified NF-κB2 as the sole overlapping candidate specifically enriched in the C5 subset (Figure 6G). Mechanistically, ChIP-qPCR and luciferase assays confirmed that the AR directly binds the *NFKB2* promoter to repress its transcriptional activity (Figure 6H–I; Figure S12A). Consequently, either pharmacological androgen blockade or shRNA-mediated *AR* depletion led to the accumulation of p52, the active form of the *NFKB2* gene product (Figure 6J). Functionally, enforced expression of p52 induced senescence, whereas inhibition of non-canonical NF-κB signaling through *NIK* silencing or treatment with the p52 inhibitor SN52 attenuated p16 accumulation and suppressed SASP production (Figure 6K–M, O–P; Figure S12B–C). Clinical validation by mIF revealed strong spatial colocalization of nuclear NF-κB2 and p16 specifically in CRPC tissues (Figure 6N; Figure S12D), supporting the translational relevance of this pathway.

We next assessed the therapeutic implications of targeting this axis.* NIK* knockdown in CAFs sensitized co-cultured tumor cells to apoptosis, effectively reversing the protective effect conferred by FAD-conditioned stroma (Figure 6Q; Figure S12E–G). In xenograft models, co-injection of NIK-deficient CAFs significantly restrained tumor growth relative to controls (Figure 6R–T), accompanied by reduced fibrosis and diminished tumor proliferation (Figure S12H–J). Consistently, mIF analysis demonstrated reduced IL-6 expression, particularly in regions enriched for p16^+^ CAFs, in the NIK-deficient group (Figure 6U). Collectively, these findings establish AR as a physiological brake on the NF-κB2/p52 pathway and demonstrate that its loss, whether driven by therapeutic pressure or intrinsic remodeling 38, activates a pro-tumorigenic senescence program that reinforces the castration-resistant niche.

### Targeting the stromal NF-κB2/p52 axis overcomes microenvironment-mediated resistance to enzalutamide

**Figure 7:**
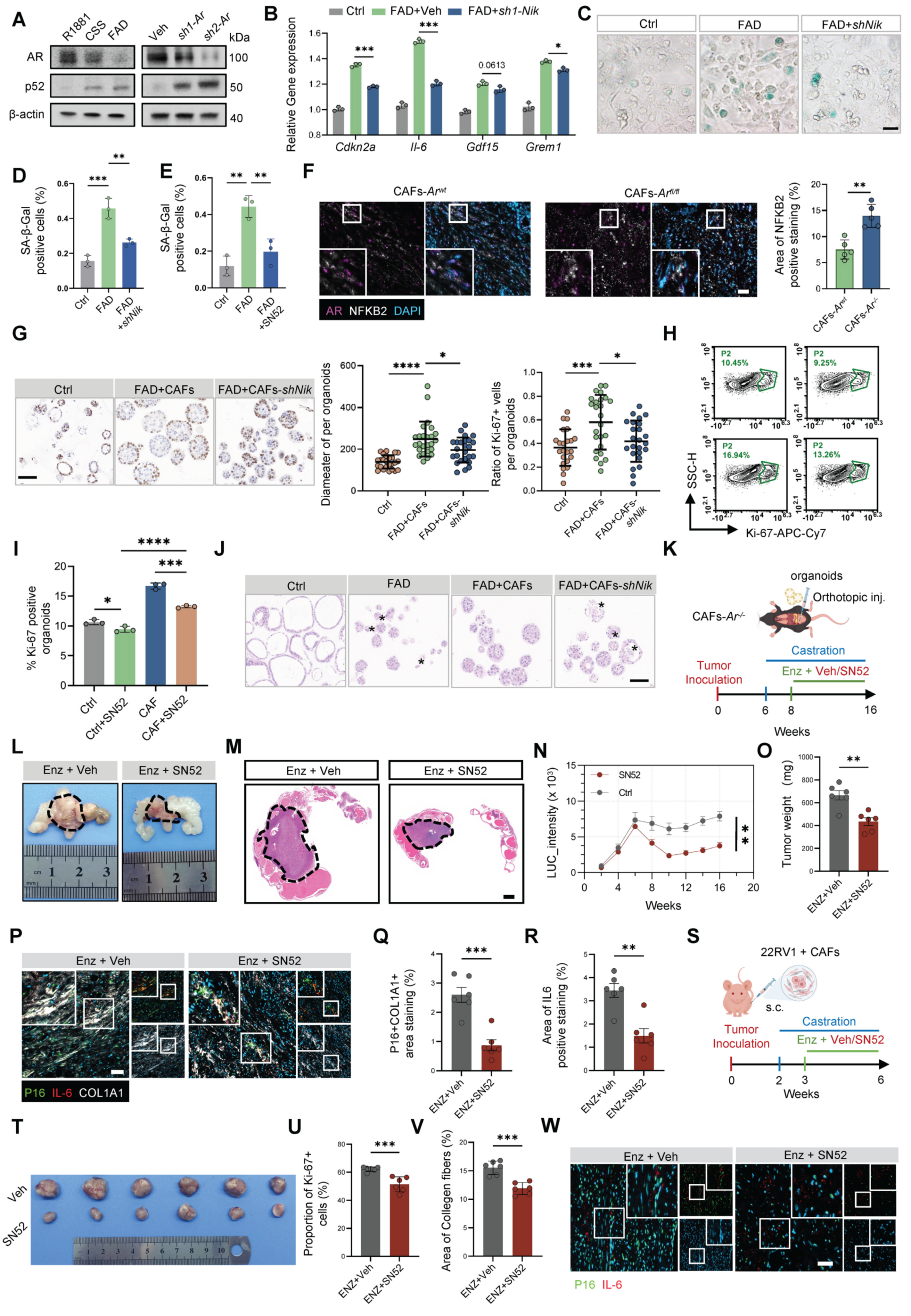
** Targeting the stromal NF-κB2/p52 axis overcomes microenvironment-mediated resistance to enzalutamide.** (A) Western blot analysis of AR and p52 expression in primary CAFs isolated from *PbsnCreERT;Pten^-/-^;Trp53^-/-^* mice with or without *Ar* deletion. (B) RT-qPCR analysis of SASP-related genes. (C-D) Representative SA-β-Gal staining (scale bar = 200 µm) (C) and quantification of SA-β-Gal positive cells (D) in CAFs transduced with control or Nik shRNA under FAD conditions. (E) Quantification of SA-β-Gal positive cells in CAFs treated with the SN52 peptide inhibitor under FAD. (F) Representative IF staining and quantification of NF-κB2 (red) in prostate tissues from *Ar^wt/wt^* and *Col1a2CreERT;Ar^-/-^* mice (scale bar = 100 µm). (G) Representative Ki-67 staining of organoids and quantification of organoid diameter and Ki-67^+^ ratio in co-cultures with CAFs expressing control or Nik shRNA. (scale bar = 100 µm). (H-I) Evaluation of SN52 treatment in organoid co-cultures including flow cytometry plots (H) and quantification of Ki-67-positive percentage (I). (J) Representative HE images showing organoid morphology. (scale bar = 100 µm). (K) Schematic of the orthotopic syngeneic model treated with Enzalutamide (Enz) and either Vehicle (Veh) or SN52. (n=6) (L-O) Longitudinal monitoring of tumor burden showing tumor photographs (L), representative HE images (scale bar = 2 mm) (M), quantification of LUC_intensity (N), and final tumor weight (O). (P-R) Multiplex IF analysis of orthotopic tumors for p16 (green), IL-6 (red), and COL1A1 (white) (scale bar = 100 µm) (P), with quantification of p16^+^COL1A1^+^ stromal cells (Q) and IL-6^+^ area (R). (S) Schematic of the subcutaneous xenograft model (22Rv1 cells + Patient-CAFs) treated with Enz and either Veh or SN52. (n=6) (T) Tumor photographs. (U) Quantification of Ki-67-positive percentage. (V) Histological quantification of collagen deposition by Masson's trichrome staining. (W) Representative mIF images showing p16 (green) and IL-6 (red) (scale bar = 100 µm). Data are presented as mean ± SEM. Statistical significance was determined using one-way ANOVA or two-way ANOVA followed by post hoc tests, or unpaired t-test for two-group comparisons. *P < 0.05, **P < 0.01, ***P < 0.001.

To validate the evolutionary conservation of this signaling axis, we employed murine models. Primary CAFs isolated from *Ppt* CRPC mice recapitulated the human phenotype, displaying pronounced p52 accumulation following *Ar* loss (Figure 7A). Although *Ar* deficiency induced a robust senescence program, inhibition of non-canonical NF-κB signaling, either through genetic *Nik* knockdown or pharmacological blockade with the p52 nuclear translocation inhibitor SN52, significantly reduced SA-β-Gal activity and suppressed expression of SASP-associated genes, including *Cdkn2a*, *Il6*, *Gdf15*, and *Grem1* (Figure 7B–D). Comparable attenuation was observed with SN52 treatment alone (Figure 7E; Figure S13A). *In vivo*,* Ar* deletion in CAFs led to a marked expansion of NF-κB2-positive stromal cells (Figure 7F), further underscoring the essential role of *Ar* in restraining stromal NF-κB2 signaling. We next assessed the translational potential of targeting this pathway. In organoid co-culture systems, disruption of p52 signaling via *Nik* knockdown (Figure 7G) or SN52 treatment significantly impaired the ability of CAFs to sustain organoid growth under FAD conditions (Figure 7H–I), as reflected by reduced organoid diameter and decreased Ki-67 index. Histological analysis further demonstrated that blockade of stromal p52 signaling induced marked structural regression, characterized by architectural disruption and reduced cellularity (Figure 7J).

Based on these findings, we hypothesized that SN52 could sensitize tumors to antiandrogen therapy. In an orthotopic model in which *Ppt* organoids were implanted into the prostates of *CAFs-Ar^-/-^* mice, bioluminescence imaging showed that SN52 combined with FAD markedly suppressed tumor progression (Figure 7K–N; Figure S13B), resulting in a substantially reduced tumor burden (Figure 7O). To determine whether this effect was mediated through attenuation of stromal senescence, mIF analysis revealed that combination therapy significantly reduced the p16^+^COL1A1^+^ senescent stromal population, accompanied by a concomitant decrease in IL-6 expression (Figure 7P–R).

In addition, immunohistochemistry demonstrated that the combined regimen effectively diminished stromal DCN expression, a marker of pro-tumorigenic inflammatory CAFs 22, indicating suppression of the inflammatory CAF transition (Figure S13C). Further supporting the translational relevance of this approach, combined SN52 and FAD treatment in xenograft models significantly inhibited tumor growth and reduced tumor weight (Figure 7S–U; Figure S13D–E), while also attenuating fibrosis (Figure 7V; Figure S13F). Consistently, IL-6 expression in the vicinity of p16^+^ senescent CAFs was markedly decreased (Figure 7W; Figure S13G). Collectively, these results demonstrate that pharmacological inhibition of the stromal NF-κB2/p52 axis is a promising therapeutic strategy to suppress treatment-induced SenCAFs and overcome castration resistance in PCa.

## Discussion

In this study, through integrative analysis of scRNA-seq data spanning PCa progression, we identified distinct microenvironmental ecotypes that evolve across the continuum of PCa progression. Most notably, we uncovered a CRPC-specific ecosystem (Ecotype 4) driven by TGF-β signaling and characterized by a collagen-dense vascular–stromal barrier that enforces immune exclusion. Crucially, our findings highlight a therapeutic paradox wherein androgen deprivation therapy indirectly stimulates the repression of stromal AR signaling. We demonstrate that AR functions as a physiological brake on the non-canonical NF-κB pathway, and its loss unleashes a pro-tumorigenic senescence program that constitutes the functional core of this resistant ecotype. Consequently, targeting this axis dismantles the stromal shield and restores therapeutic sensitivity, establishing the stromal AR-p52 axis as a critical vulnerability in advanced disease.

The concept of the TME as an organized ecosystem in which distinct cellular states co-evolve has gained increasing recognition, with recent studies defining recurrent ecotypes that predict therapeutic response across multiple malignancies 12, 39. However, the evolutionary dynamics of TME ecotypes under sustained androgen deprivation, particularly during the transition to CRPC, have remained insufficiently characterized. Our findings address this gap by mapping the trajectory of TME ecotypes throughout PCa progression. Although HSPC displays heterogeneous microenvironmental states, ranging from adaptive immune–enriched communities (Ecotype 1) to macrophage-dominated myeloid niches (Ecotype 3), the overall landscape remains immunologically “cold,” likely reflecting dominant myeloid-driven immunosuppression within Ecotype 3.

Upon transition to castration resistance, these heterogeneous microenvironmental states converge toward a stromal-dominant configuration defined as Ecotype 4. This resistance-associated niche is characterized by the coordinated assembly of COL4A1^+^ capillary endothelial cells, POSTN^+^ pericytes, SPP1^+^ tumor-associated macrophages (TAM), and C4_CAFs. A central architectural hallmark of this niche is pathological remodeling of the vascular basement membrane. Although angiogenesis is a common feature of malignancy, the CRPC-specific enrichment of COL4A1 within capillary endothelial cells indicates a hypertrophic, matrix-producing phenotype rather than functional vascular normalization 40-43. In contrast to physiological angiogenesis, this excessive collagen deposition, together with the tight ensheathment by THY1^+^POSTN^hi^ pericytes, forms a rigid and impermeable vascular sheath 44. By altering the mechanical properties of the perivascular space, this collagen-reinforced barrier restricts extravasation of cytotoxic T cells. Consequently, the immune-desert phenotype in CRPC is sustained not only by suppressive signaling but also by structural fortification that physically shields the niche.

Consistent with emerging evidence implicating TGF-β–mediated stromal plasticity in therapeutic resistance 10, our findings identify TGF-β signaling as an extrinsic coordinator of this multicellular architecture. Through synchronized reprogramming of endothelial, perivascular, and fibroblastic compartments, in concert with SPP1^+^ macrophages, TGF-β promotes formation of an immune-privileged fortress. These results suggest that the limited efficacy of checkpoint blockade in CRPC may arise from TGF-β–driven architectural exclusion rather than solely from tumor-intrinsic immune evasion, providing a structural framework for understanding resistance to immunotherapy in this setting.

Our study further delineates the multi-layered complexity of the CRPC microenvironment. In addition to the vascular barrier described above, a central discovery is the identification of an AR-negative, senescent C5_CAF subset, referred to as SenCAFs, which actively drives maladaptive stromal evolution under ADT. Although conventional ADT primarily disrupts AR ligand binding, we observed near-complete loss of stromal AR expression at both the transcript and protein levels within the CRPC niche. Consistent with prior evidence that tumor-derived signals can transcriptionally suppress stromal AR 38, this finding suggests a tumor-driven “hijacking” mechanism. By extinguishing the homeostatic AR restraint 45, therapy-adapted tumor cells appear to reprogram surrounding fibroblasts into a pro-tumorigenic senescent state that amplifies survival signaling. Trajectory analysis further indicates that SenCAFs represent a transitional hub rather than a terminal lineage state, serving as an intermediate that bridges toward the dense, matrix-producing C4 myCAF phenotype. This positioning implies that stromal senescence functions as a pivotal inflection point in TME remodeling, initiating a feed-forward cascade that culminates in persistent fibrosis and structural reinforcement of the resistant niche.

Recent studies have highlighted the differentiation of stromal senescence, including the identification of a p21-positive CAF subset that promotes immune suppression 46. Intriguingly, our data identify p16, rather than p21, as the dominant senescence marker within the AR-depleted stromal population under ADT, underscoring the context-specific nature of senescence programs. Although Zhou et al. demonstrated the efficacy of senolytic therapy with ABT-263 (Navitoclax) to eliminate senescent CAFs, our findings point to a complementary strategy. Instead of exclusively targeting senescent cells after their establishment, a more precise intervention may involve disrupting the upstream molecular events that commit fibroblasts to this trajectory following AR loss, thereby preventing formation of the senescent niche at its inception.

Mechanistically, we map this regulatory switch to the AR–NF-κB2 axis. Our data demonstrate that AR directly represses NF-κB2 transcription, functioning as a physiological constraint on non-canonical NF-κB signaling. While the canonical p65 complex is widely regarded as a central regulator of SASP 37, 47, our results reveal a specific dependence on the p52 pathway during ADT-driven stromal remodeling, consistent with prior reports linking nuclear p52 to mesenchymal cell senescence 48. We therefore identify the “AR-off” state in CAFs as a critical upstream event that unleashes NF-κB2-mediated senescence. Importantly, given that aberrant NIK/p52 signaling also contributes to tumor-intrinsic resistance, including induction of AR splice variants 49, targeting this axis may offer a dual-hit strategy. Such an approach could simultaneously dismantle the senescence-associated stromal barrier and suppress tumor cell survival pathways, thereby addressing both the “soil” and the “seed” of castration resistance.

In conclusion, our study defines the principal microenvironmental ecotypes that emerge across distinct stages of PCa progression and delineates the dominant cellular constituents that shape the castration-resistant niche. We demonstrate that although ADT effectively targets tumor cells, it paradoxically induces adaptive stromal remodeling, leading to the formation of a fibrotic vascular barrier and, critically, p52-driven CAF senescence. This remodeling reveals a fundamental limitation of current therapeutic strategies, which largely neglect the dynamic contribution of the non-malignant stroma to disease progression. By characterizing this protective niche and identifying the stromal AR–non-canonical NF-κB2–p52 axis as a central regulatory mechanism, we provide a mechanistic framework for therapeutic intervention. Ultimately, our findings support a combinatorial approach integrating p52 inhibition with androgen deprivation to dismantle the stromal barrier and restore therapeutic sensitivity within the tumor ecosystem.

## Supplementary Material

Supplementary figures.

Supplementary table 1.

Supplementary table 2.

Supplementary table 3.

Supplementary table 4.

Supplementary table 5.

Supplementary table 6.

Supplementary table 7.

## Data Availability

Published datasets used in this study are available through cBioPortal or GEO as indicated in the Table S1. The raw RNA-seq datasets generated in this research are available at Gene Expression Omnibus repository (GSE328022). All bioinformatic processing was conducted using standard open-source algorithms. Further details or metadata necessary to reproduce the analysis pipelines are available from the authors upon reasonable request.

## Abbreviations

AR: androgen receptor
TME: tumor microenvironment
ADT: androgen deprivation therapy
PCa: prostate cancer
CRPC: castration-resistant prostate cancer
mCRPC: metastatic castration-resistant prostate cancer
HSPC: hormone-sensitive prostate cancer
mHSPC: metastatic hormone-sensitive prostate cancer
CAFs: cancer-associated fibroblasts
myCAFs: myofibroblastic cancer-associated fibroblasts
SenCAFs: senescent cancer-associated fibroblasts
HVGs: highly variable genes
DEGs: differentially expressed genes
Ro/e: ratio of observed to expected
RSS: regulon specificity scores
ST: spatial transcriptomics
GSEA: gene set enrichment analysis
TF: transcription factor
FAD: full androgen deprivation
CSS: charcoal-stripped serum
CM: conditioned medium
ChIP: chromatin immunoprecipitation
SA-β-gal: senescence-associated β-gal
H&E: hematoxylin and eosin
mIF: multiplex immunofluorescence
UMAP: uniform manifold approximation and projection
TLS: tertiary lymphoid structures
ECM: extracellular matrix
TAM: tumor-associated macrophages
GS: Gleason scores
OR: odds ratio
Enz: enzalutamide
