# Supplementary material for: Therapeutic pressure drives the evolution of a protective ecotype characterized by AR-loss-induced senescence in prostate cancer
Source: Theranostics. 2026 May 11;16(12):6803–26. doi: 10.7150/thno.134940 (PMC13232435; doi:10.7150/thno.134940)
Supplement: Supplementary file 1 — Supplementary figures. [file thnov16p6803s1.pdf]

Supplementary

Figure S1

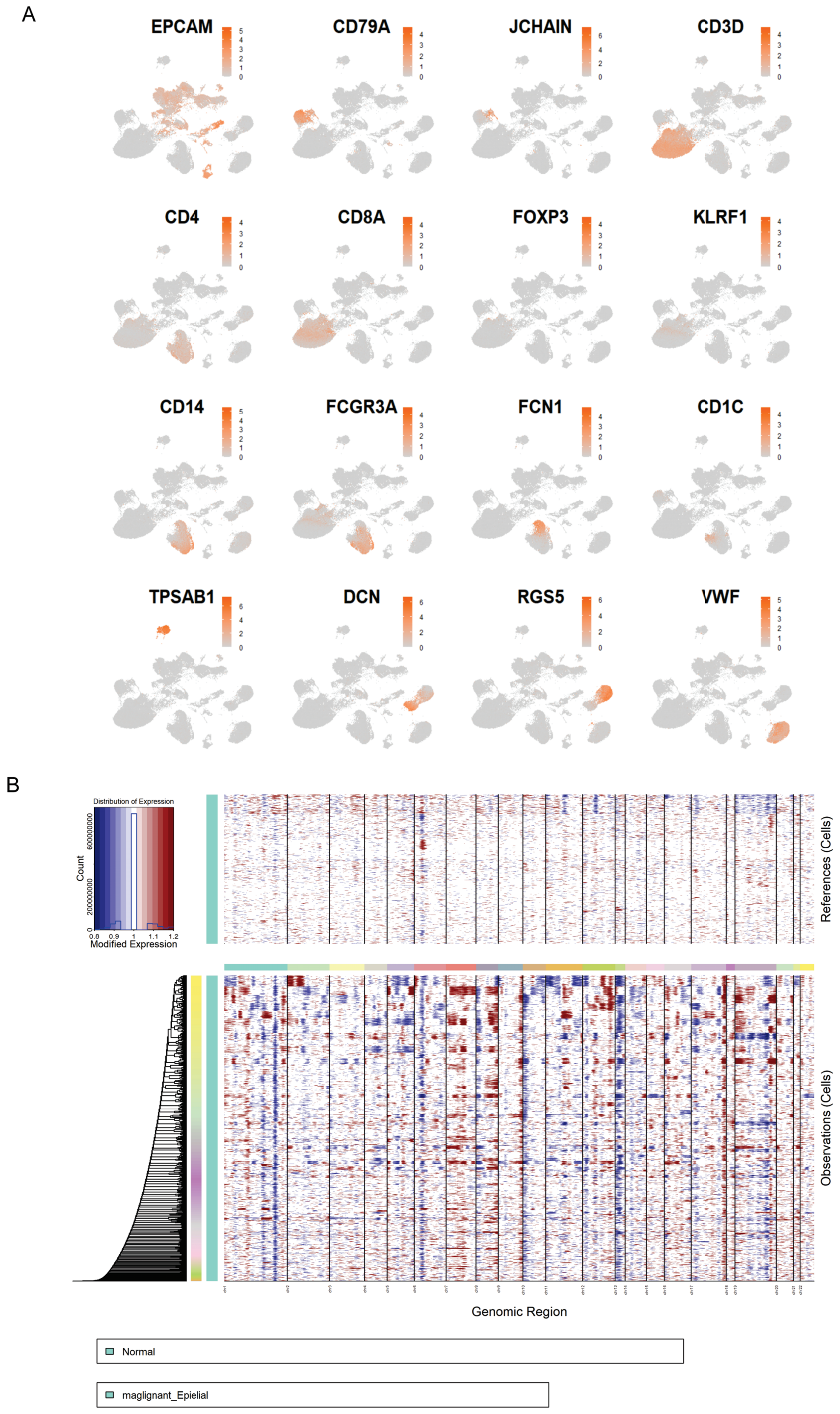

Figure S1. Global atlas and data integration quality control (Related to Figure 1).

A, UMAP visualization of marker gene expression across major cell types. **B,** Chromosomal landscape of inferred CNVs from scRNA-seq data, distinguishing malignant cell subclusters. T cells were used as the reference. Red indicates chromosomal amplifications, and blue denotes deletions.

Figure S2

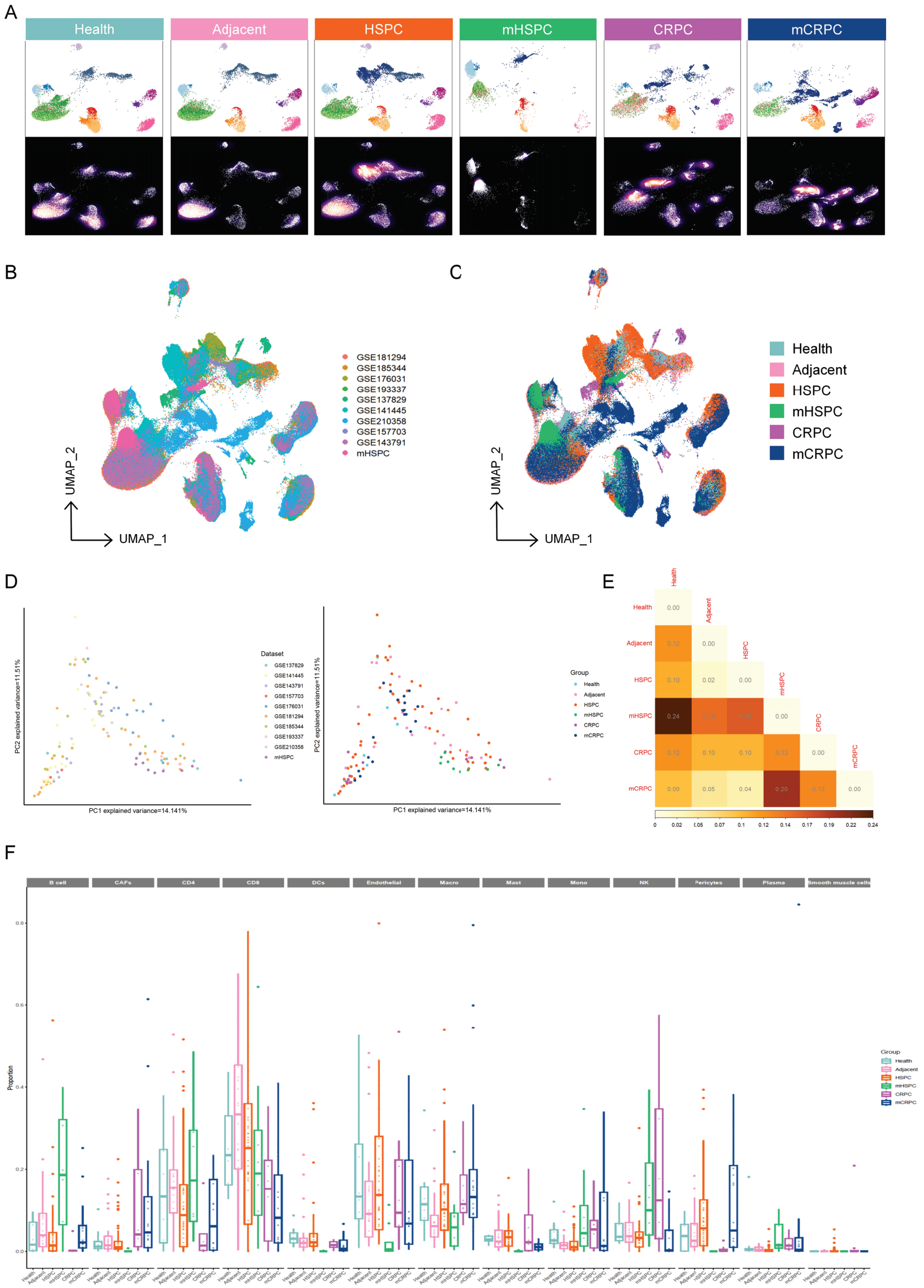

A
Health
Adjacent
HSPC
mHSPC
CRPC
mCRPC
B
GSE181294
GSE185344
GSE176031
GSE193337
GSE137829
GSE141445
GSE210358
GSE157703
GSE143791
mHSPC
UMAP_2
UMAP_1
C
Health
Adjacent
HSPC
mHSPC
CRPC
mCRPC
UMAP_2
UMAP_1
D
PC2 explained variance=11.51%
PC1 explained variance=14.141%
Dataset
Group
E
F
Proportion
B cell
CAFs
CD4
CD8
DCs
Endothelial
Macro
Mast
Mono
NK
Plasma
Smooth muscle cells

Figure S2. Annotation of major cell lineages in the PCa microenvironment (Related to Figure 1).

A, The UMAP plot displays the downsampling analysis shown indicates that the results are reproducible and are not affected by the total number of cells from each tissue type. **B,** UMAP showing cell clustering colored by datasets. **C,** UMAP showing cell clustering colored by the tissue origins. **D,** PCA plots showing sample clustering based on cell subset abundance, colored by datasets (left) and tissue type(right). **E,** Heatmap showing the distances between each two tissue types. **F,** Boxplots showing all TME cell subclusters proportion in different tissues.

Figure S3

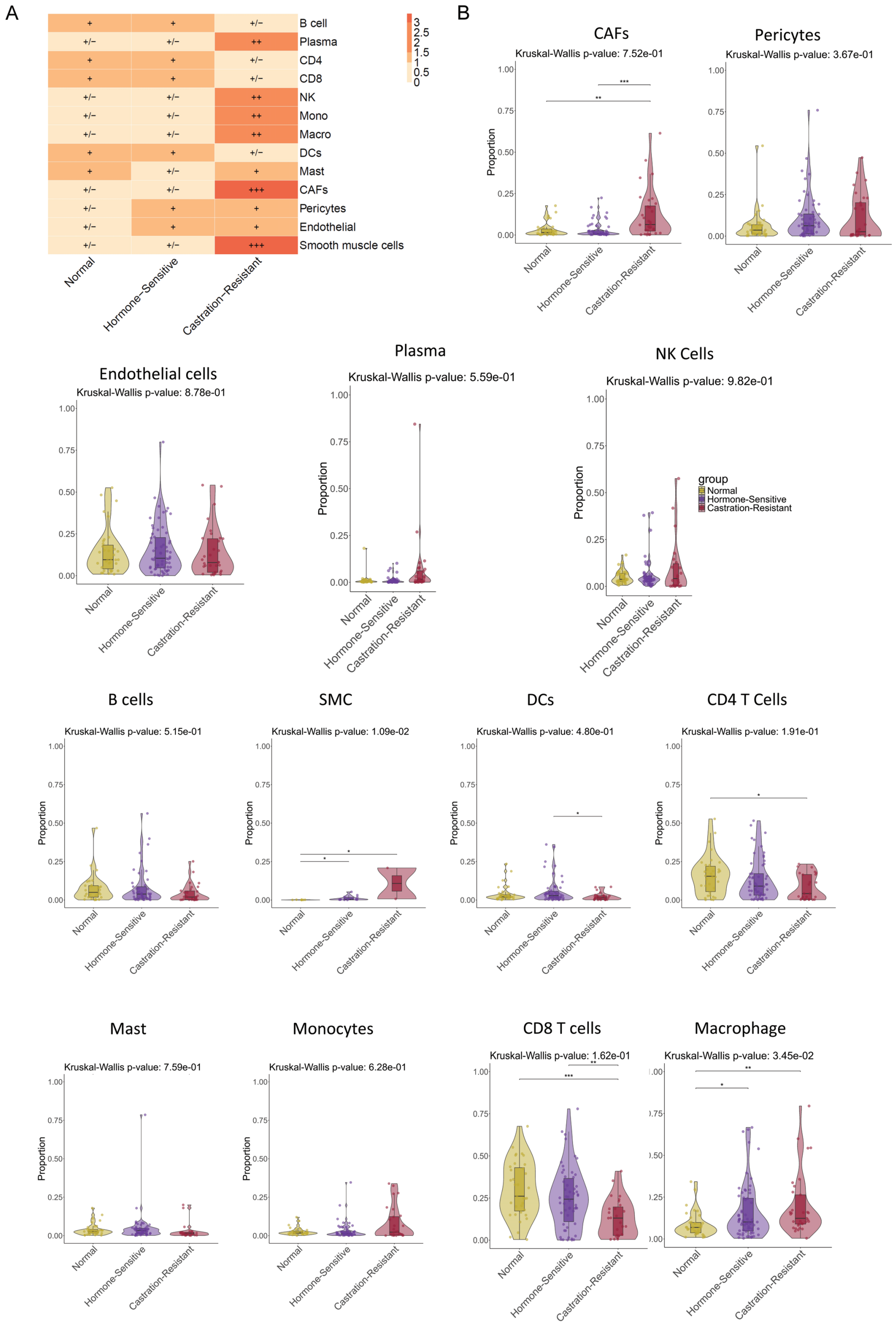
A
B cell
Plasma
CD4
CD8
NK
Mono
Macro
DCs
Mast
CAFs
Pericytes
Endothelial
Smooth muscle cells
Normal
Hormone−Sensitive
Castration−Resistant
B
CAFs
Kruskal-Wallis p-value: 7.52e-01
Pericytes
Kruskal-Wallis p-value: 3.67e-01
Endothelial cells
Kruskal-Wallis p-value: 8.78e-01
Plasma
Kruskal-Wallis p-value: 5.59e-01
NK Cells
Kruskal-Wallis p-value: 9.82e-01
group
Normal
Hormone-Sensitive
Castration-Resistant
B cells
Kruskal-Wallis p-value: 5.15e-01
SMC
Kruskal-Wallis p-value: 1.09e-02
DCs
Kruskal-Wallis p-value: 4.80e-01
CD4 T Cells
Kruskal-Wallis p-value: 1.91e-01
Mast
Kruskal-Wallis p-value: 7.59e-01
Monocytes
Kruskal-Wallis p-value: 6.28e-01
CD8 T cells
Kruskal-Wallis p-value: 1.62e-01
Macrophage
Kruskal-Wallis p-value: 3.45e-02
Proportion

Figure S3. Transcriptional heterogeneity of epithelial and stromal subsets (Related to Figure 1 and Figure 2).

A, Heatmap displaying the prevalence of major cell types in each group. (Ro/e) **B,** Violin plots and bar plots showing the proportion of each major cell type in the normal, hormone-sensitive, and castration-resistant groups.

Figure S4

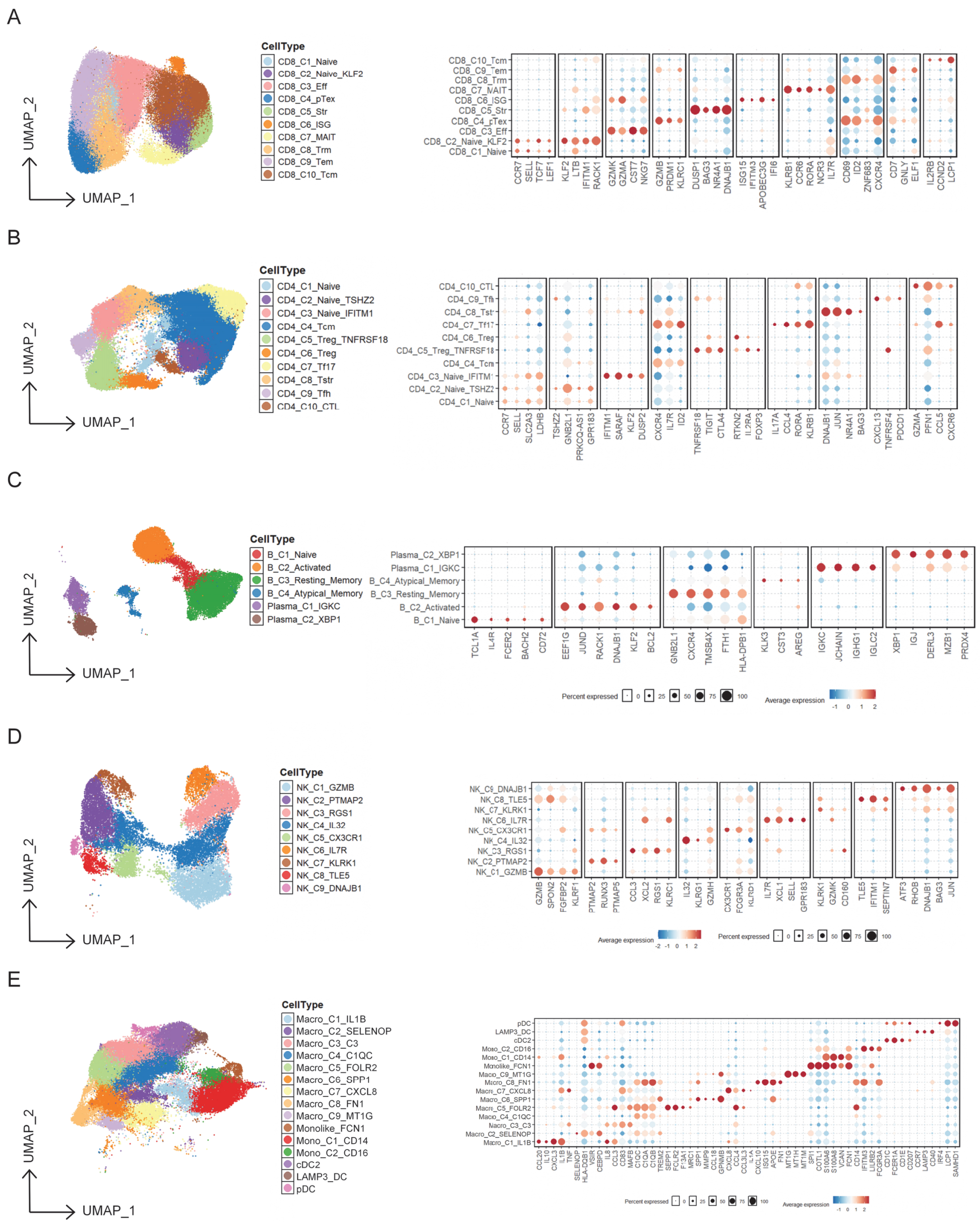

Figure S4. Functional Characterization and Metabolic States of CD8$^+$ T Cells (Related to Figure 2).

A-E, UMAP and dot plots showing T cells, B cells, NK cells, macrophages, and their marker genes.

Figure S5

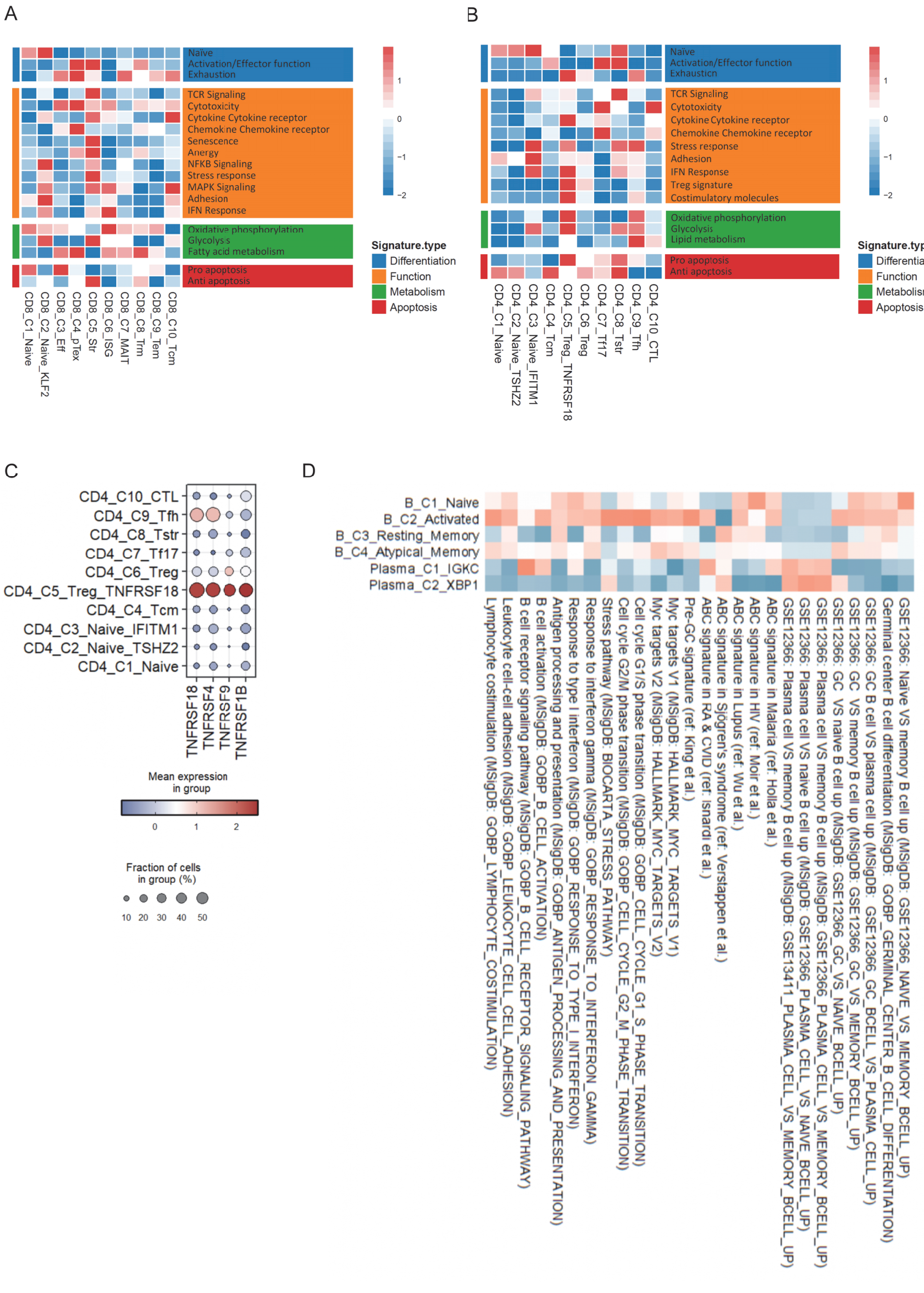

Figure S5. Phenotypic diversity and regulatory features of CD4$^+$ T cells (Related to Figure 2). A-B, Heatmap illustrating expression of curated gene signatures across T cell clusters. Heatmap was generated based on the scaled gene signature scores. **C,** Bubble plot showing the expression of key genes in CD4 T cell subsets. **D,** Heatmap showing expression of gene signatures in B cell subsets.

Figure S6

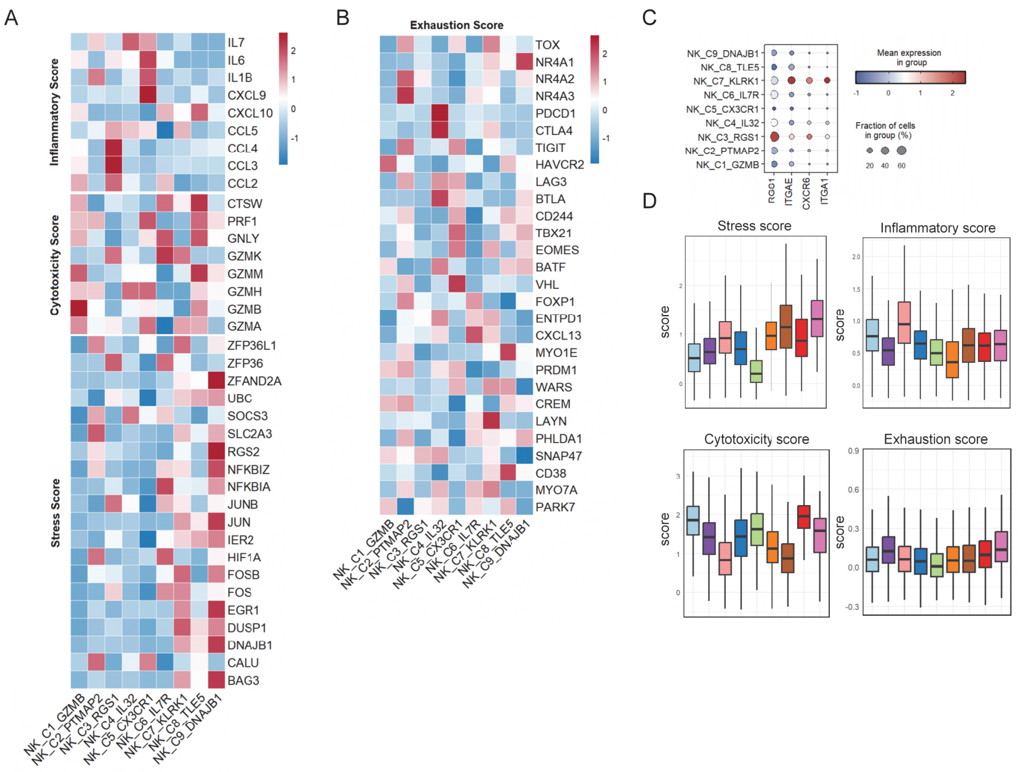

Figure S6. Quantitative assessment of immune functional states across disease stages (Related to Figure 2).

A-B, Heatmap showing the expression of corresponding genes used to define the functional scores. Color indicates the Z score scaled gene expression levels. **C,** Bubble plot showing the expression of key genes in NK cell subsets. **D,** Bar plots showing the functional gene set scores for each NK cell subclusters. **E,** Heatmap showing tissue enrichment for each cell subcluster, as determined by the Ro/e score.

Figure S7

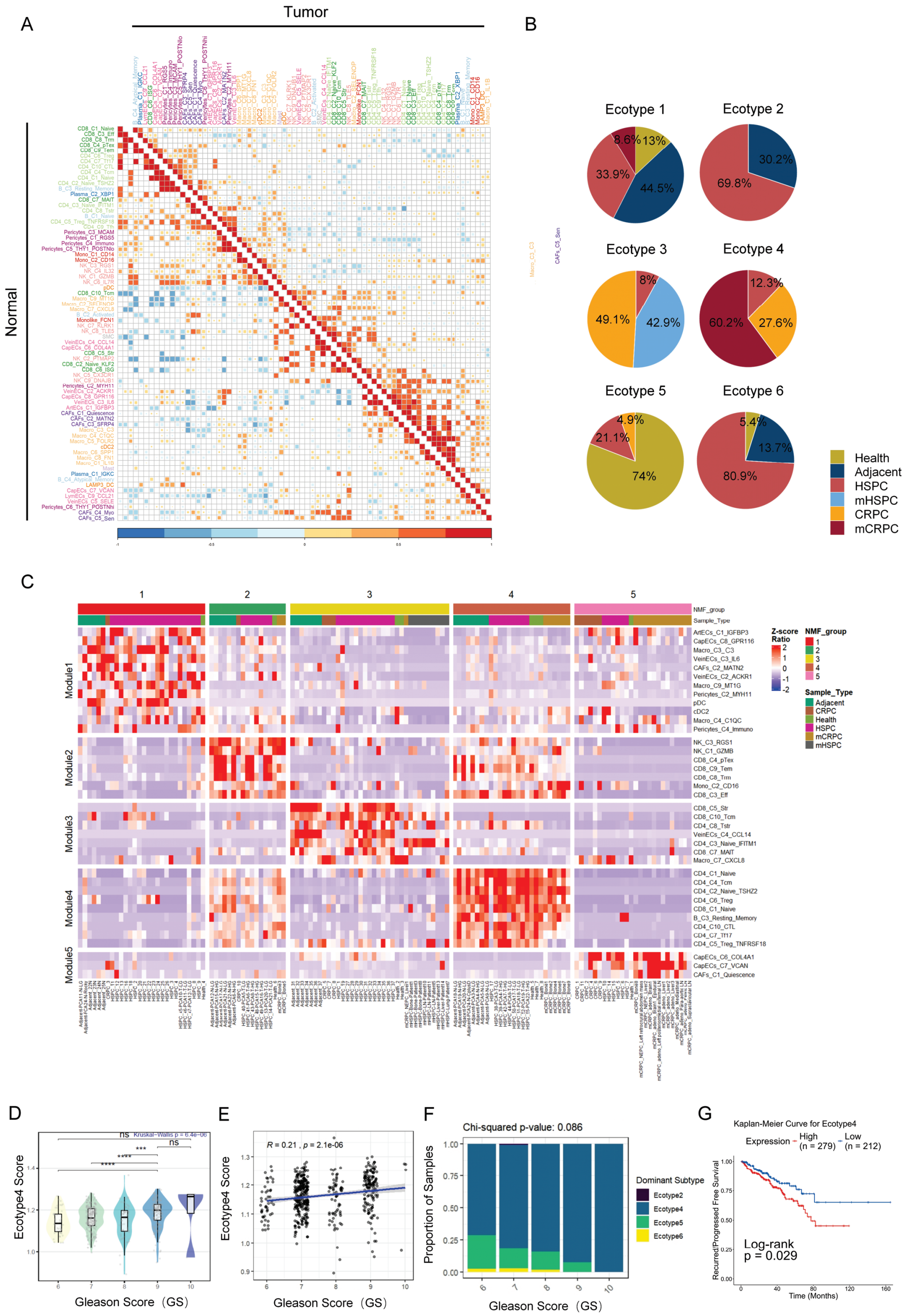
A
Tumor
Normal
B
Ecotype 1
8.6%
13%
33.9%
44.5%
Ecotype 2
30.2%
69.8%
Ecotype 3
8%
49.1%
42.9%
Ecotype 4
12.3%
60.2%
27.6%
Ecotype 5
4.9%
21.1%
74%
Ecotype 6
5.4%
13.7%
80.9%
Health
Adjacent
HSPC
mHSPC
CRPC
mCRPC
C
1
2
3
4
5
NMF_group
Sample_Type
Module1
Module2
Module3
Module4
Module5
ArtECs_C1_IGFBP3
CapECs_C8_GPR116
Macro_C3_C3
VeinECs_C3_IL6
CAFs_C2_MATN2
VeinECs_C2_ACKR1
Macro_C9_MT1G
Pericytes_C2_MYH11
pDC
cDC2
Macro_C4_C1QC
Pericytes_C4_Immuno
NK_C3_RGS1
NK_C1_GZMB
CD8_C4_pTex
CD8_C9_Tem
CD8_C8_Trm
Mono_C2_CD16
CD8_C3_Eff
CD8_C5_Str
CD8_C10_Tcm
CD4_C6_Tstr
VeinECs_C4_CCL14
CD4_C3_Naive_IFITM1
CD8_C7_MAIT
Macro_C7_CXCL8
CD4_C1_Naive
CD4_C4_Tcm
CD4_C2_Naive_TSHZ2
CD4_C6_Treg
CD8_C1_Naive
B_C3_Resting_Memory
CD4_C10_CTL
CD4_C7_Th17
CD4_C5_Treg_TNFRSF18
CapECs_C6_COL4A1
CapECs_C7_VCAN
CAFs_C1_Quiescence
Z-score
Ratio
NMF_group
Sample_Type
Adjacent
CRPC
Health
HSPC
mCRPC
mHSPC
D
Ecotype4 Score
Kruskal-Wallis
ns
Gleason Score (GS)
E
R = 0.21, p = 2.1e-06
Ecotype4 Score
Gleason Score (GS)
F
Chi-squared p-value: 0.086
Proportion of Samples
Dominant Subtype
Ecotype2
Ecotype4
Ecotype5
Ecotype6
Gleason Score (GS)
G
Kaplan-Meier Curve for Ecotype4
Expression
High (n = 279)
Low (n = 212)
Recurred/Progressed Free Survival
Log-rank
p = 0.029
Time (Months)

Figure S7. Characterization of TME ecotypes and clinical relevance (Related to Figure 3)

A, Heatmap showing correlation coefficients between cell subclusters in tumor (right) and normal (left) groups. (Spearman's correlation test) **B,** Pie charts showing the sample type composition within each ecotype. **C,** NMF classification revealed patients with five distinct TIME subtypes, each enriched with distinct cell types. Values in heatmap indicate the relative proportion of each cell type in TME cells. **D–F,** Correlation analysis between Ecotype 4 scores and Gleason Scores (GS) in the TCGA-PRAD cohort. A significant overall difference was found among GS groups (Kruskal-Wallis test, $p = 6.4e-06$). Statistical significance: ns, not significant; ***, $p < 0.001$; ****, $p < 0.0001$. **G,** Kaplan-Meier survival curves depicting recurrence/progression-free survival in the TCGA cohort, stratified by high versus low Ecotype 4 scores.

Figure S8

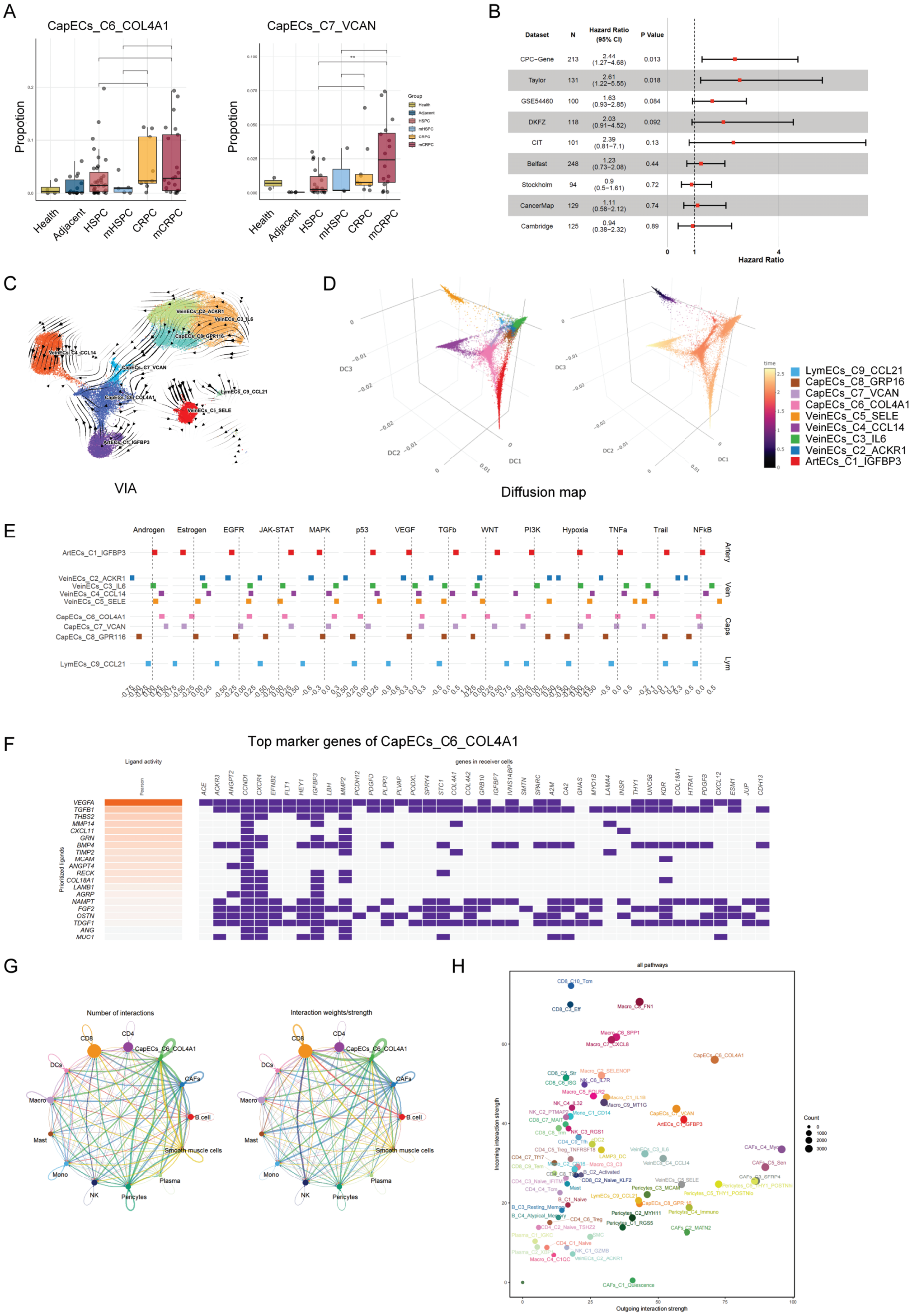

A
CapECs_C6_COL4A1
CapECs_C7_VCAN
Propotion
Health
Adjacent
HSPC
mHSPC
CRPC
mCRPC
Group
B
Dataset
N
Hazard Ratio (95% CI)
P Value
CPC−Gene 213 2.44 (1.27−4.68) 0.013
Taylor 131 2.61 (1.22−5.55) 0.018
GSE54460 100 1.63 (0.93−2.85) 0.084
DKFZ 118 2.03 (0.91−4.52) 0.092
CIT 101 2.39 (0.81−7.1) 0.13
Belfast 248 1.23 (0.73−2.08) 0.44
Stockholm 94 0.9 (0.5−1.61) 0.72
CancerMap 129 1.11 (0.58−2.12) 0.74
Cambridge 125 0.94 (0.38−2.32) 0.89
Hazard Ratio
C
VIA
D
Diffusion map
DC1
DC2
DC3
time
LymECs_C9_CCL21
CapECs_C8_GRP16
CapECs_C7_VCAN
CapECs_C6_COL4A1
VeinECs_C5_SELE
VeinECs_C4_CCL14
VeinECs_C3_IL6
VeinECs_C2_ACKR1
ArtECs_C1_IGFBP3
E
Androgen
Estrogen
EGFR
JAK-STAT
MAPK
p53
VEGF
TGFb
WNT
PI3K
Hypoxia
TNFa
Trail
NFkB
ArtECs_C1_IGFBP3
VeinECs_C2_ACKR1
VeinECs_C3_IL6
VeinECs_C4_CCL14
VeinECs_C5_SELE
CapECs_C6_COL4A1
CapECs_C7_VCAN
CapECs_C8_GPR116
LymECs_C9_CCL21
Artery
Vein
Caps
Lym
F
Top marker genes of CapECs_C6_COL4A1
Ligand activity
genes in receiver cells
Prioritized ligands
VEGFA
TGFB1
THBS2
MMP14
CXCL11
GRN
BMP4
TIMP2
MCAM
ANGPT4
RECK
COL18A1
LAMB1
AGRP
NAMPT
FGF2
OSTN
TDGF1
ANG
MUC1
G
Number of interactions
Interaction weights/strength
CD8
CD4
CapECs_C6_COL4A1
CAFs
B cell
Smooth muscle cells
Plasma
Pericytes
NK
Mono
Mast
Macro
DCs
H
all pathways
Outgoing interaction strength
Incoming interaction strength
Count

Figure S8. Characterization of the CapECs_C6_COL4A1 Subpopulation (Related to Figure 4).

A, Bar plots showing the proportion of endothelial cell (EC) subclusters across disease stages. **B,** Forest plot summarizing the hazard ratios (HR) of the CapECs_C6_COL4A1 gene signature for biochemical recurrence-free survival across multiple independent PCa cohorts. **C–D,** Trajectory inference using VIA (C) and Diffusion Map (D) algorithms, identifying CapECs_C6_COL4A1 as a terminally differentiated state. **E,** PROGENy pathway activity analysis of EC subclusters. **F,** NicheNet analysis predicting top upstream ligands regulating the CapECs_C6_COL4A1 transcriptional program. **G–H,** CellChat analysis showing the dominant signaling roles of CapECs_C6_COL4A1 as a hub in the TME communication network.

Figure S9

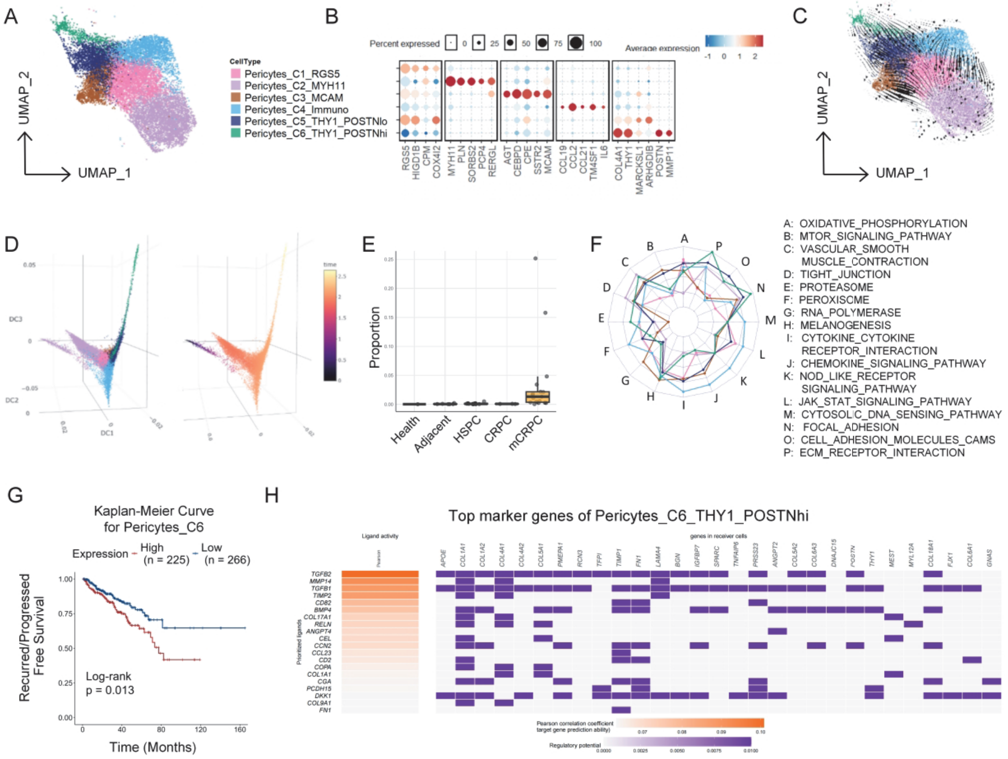

Figure S9. Characterization of Pericytes_C6_THY1_POSTNhi and the vascular-stromal barrier (Related to Figure 4).

A-B, UMAP visualization of pericytes subclusters. Dot plot illustrating the expression of marker genes across endothelial cell subclusters. **C-D,** Trajectory inference using VIA (C) and Diffusion Map (D) algorithms, identifying Pericytes_C6_THY1_POSTNhi as a terminally differentiated state. **E,** Bar plots showing the proportion of Pericytes_C6_THY1_POSTNhi subclusters across disease stages. **F,** Radar chart showcasing KEGG pathway enrichment analysis for each pericytes subcluster. **G,** Kaplan-Meier survival analysis of recurrence/progression-free survival based on the Pericytes_C6_THY1_POSTNhi gene signature. **H,** NicheNet analysis predicting top upstream ligands regulating the Pericytes_C6_THY1_POSTNhi transcriptional program.

Figure S10

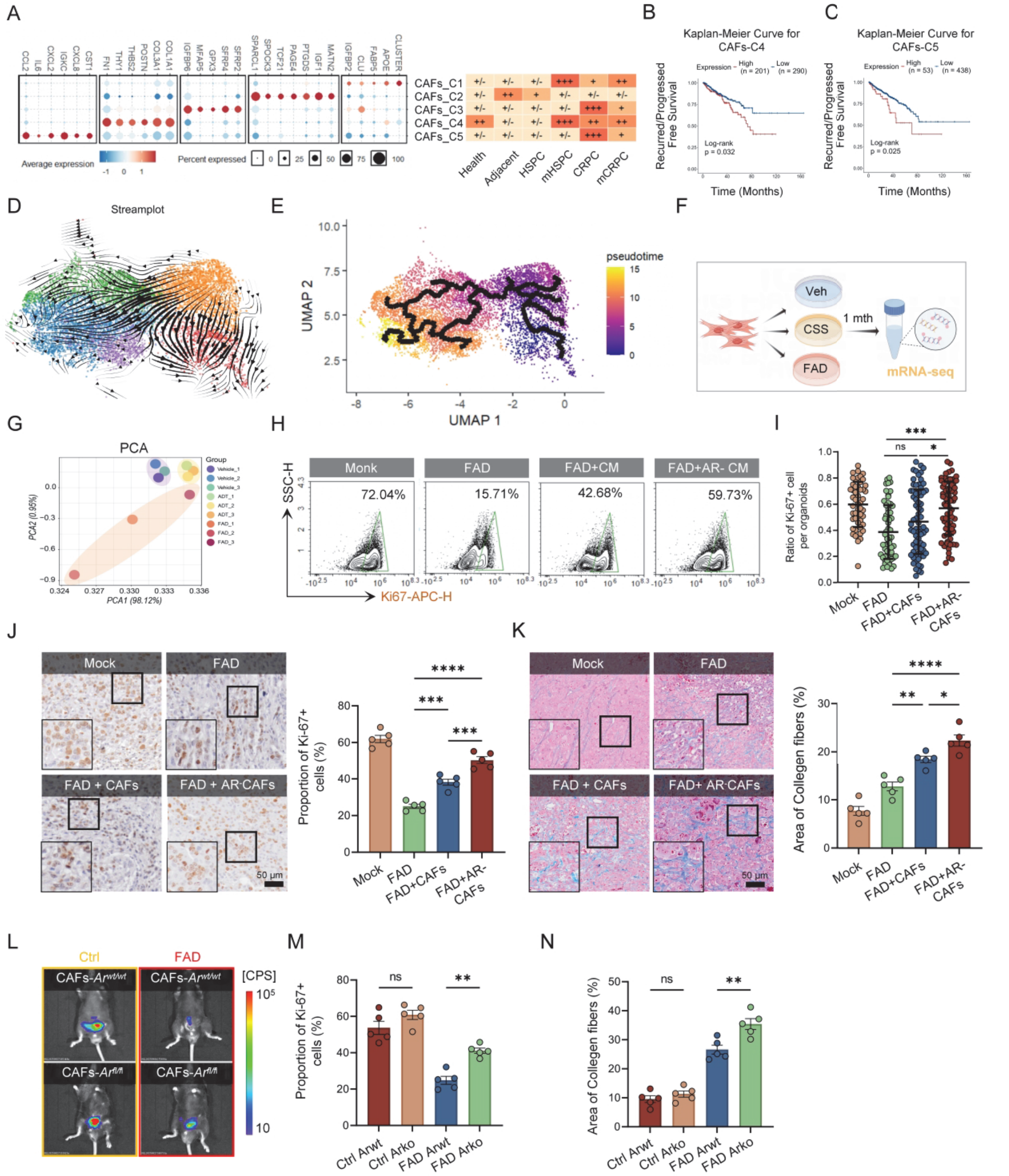

Figure S10. Functional validation of AR loss in CAFs in vitro and in vivo (Related to Figure 5).

A, Dot plot illustrating the expression of marker genes across CAFs, and heatmap showing Ro/e analysis across disease stages. **B-C,** Kaplan-Meier survival analysis for CAFs-C4 and CAFs-C5 signatures in TCGA-PRAD cohort. **D-E,** Trajectory analysis illustrating the transition from senescent-like CAFs-C5 to matrix-producing CAFs-C4 by Monocle3. **F-G,** In vitro validation related to Fig5.D. Schematic diagram (F) and Principal Component Analysis (PCA) plot of bulk RNA-seq data from F (G) . **H,** Representative flow cytometry plots of Ki-67 related to Fig5.H. **I,** Quantification of the proportion of Ki-67^{+} cells in organoids from co-culture assays related to Fig5.L. **J,** Representative images (left) and quantification (right) of Ki-67 IHC staining in xenograft tumors related to Fig5.M. (scale bar = 50 μm). **K,** Representative images (left) and

quantification (right) of Masson's trichrome staining for collagen deposition related to Fig5.M. (scale bar = 50 μm). **L,** Representative bioluminescence imaging of mice showing tumor burden related to Fig5.P. **M,** Quantification of Ki-67^{+} cells corresponding to the representative images shown in Fig. 5S. **N,** Quantification of collagen fiber area corresponding to the representative images shown in Fig. 5T. Data are presented as mean ± SEM. Statistical significance was determined using one-way ANOVA or two-way ANOVA followed by post hoc tests, and unpaired t-test for two-group comparisons. **P* < 0.05,***P* < 0.01, ****P* < 0.001.

Figure S11

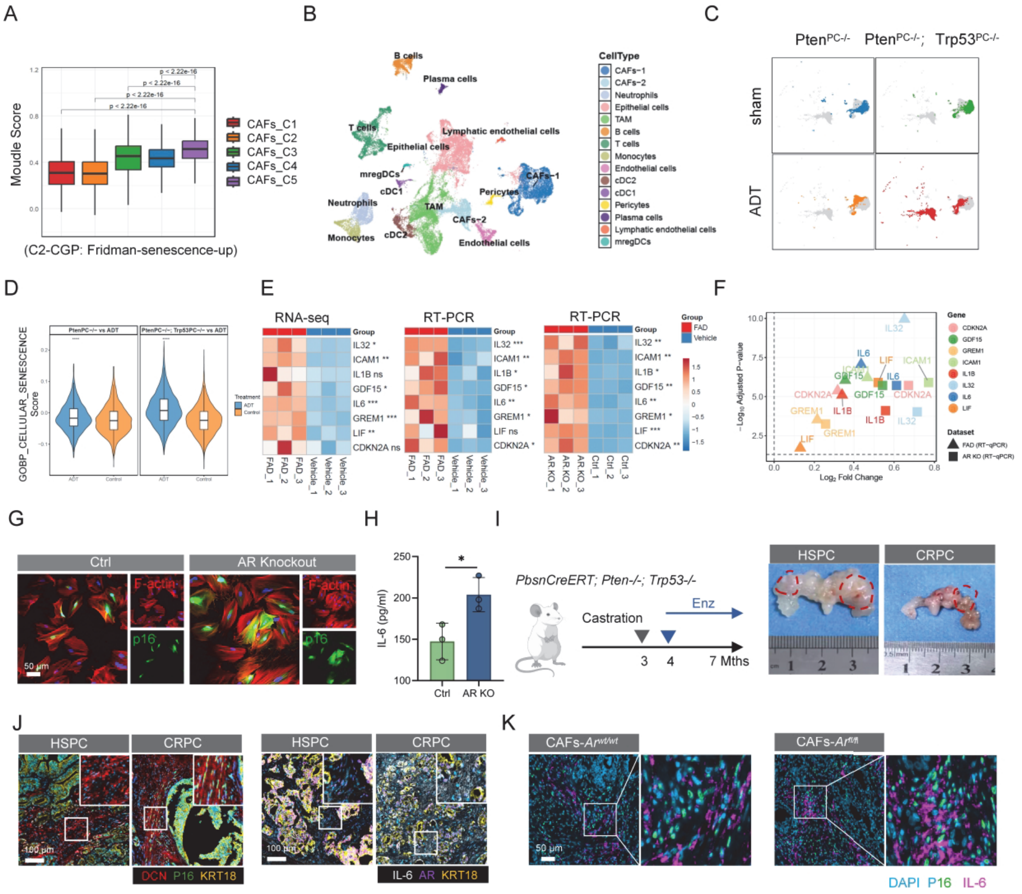

Figure S11. AR loss drives a senescence program in CAFs (Related to Figure 6).

A, Cellular senescence scores of CAFs. **B-D,** Validation in a murine scRNA-seq dataset. UMAP (B), proportion analysis (C), and senescence scores (D) of CAF subsets in HSPC vs. CRPC mouse models. **E,** Heatmap of differentially expressed senescence-associated genes in primary human CAFs treated with FAD or AR-knockout (RNA-seq and RT-PCR). **F,** RT-qPCR validation of selected senescence genes (e.g., CDKN2A, IL6) in CAFs. **G-H,** Representative mIF images (scale bar = 50 μm) (G) and ELISA for IL-6 secretion (H) in CAFs upon FAD treatment or AR depletion. **I-J,** Schematic, tumor photographs (I) and representative mIF images (scale bar = 100 μm) (J) of DCN, P16, IL-6, KRT18 and AR staining in *PbsnCreERT;Pten-/-;Trp53-/-* mouse tumors. K, Representative IF staining of P16 and IL6 in *Col1a2CreERT;Ar$^{fl/fl}$* versus wild-type mouse prostates. (scale bar = 50 μm).

Figure S12

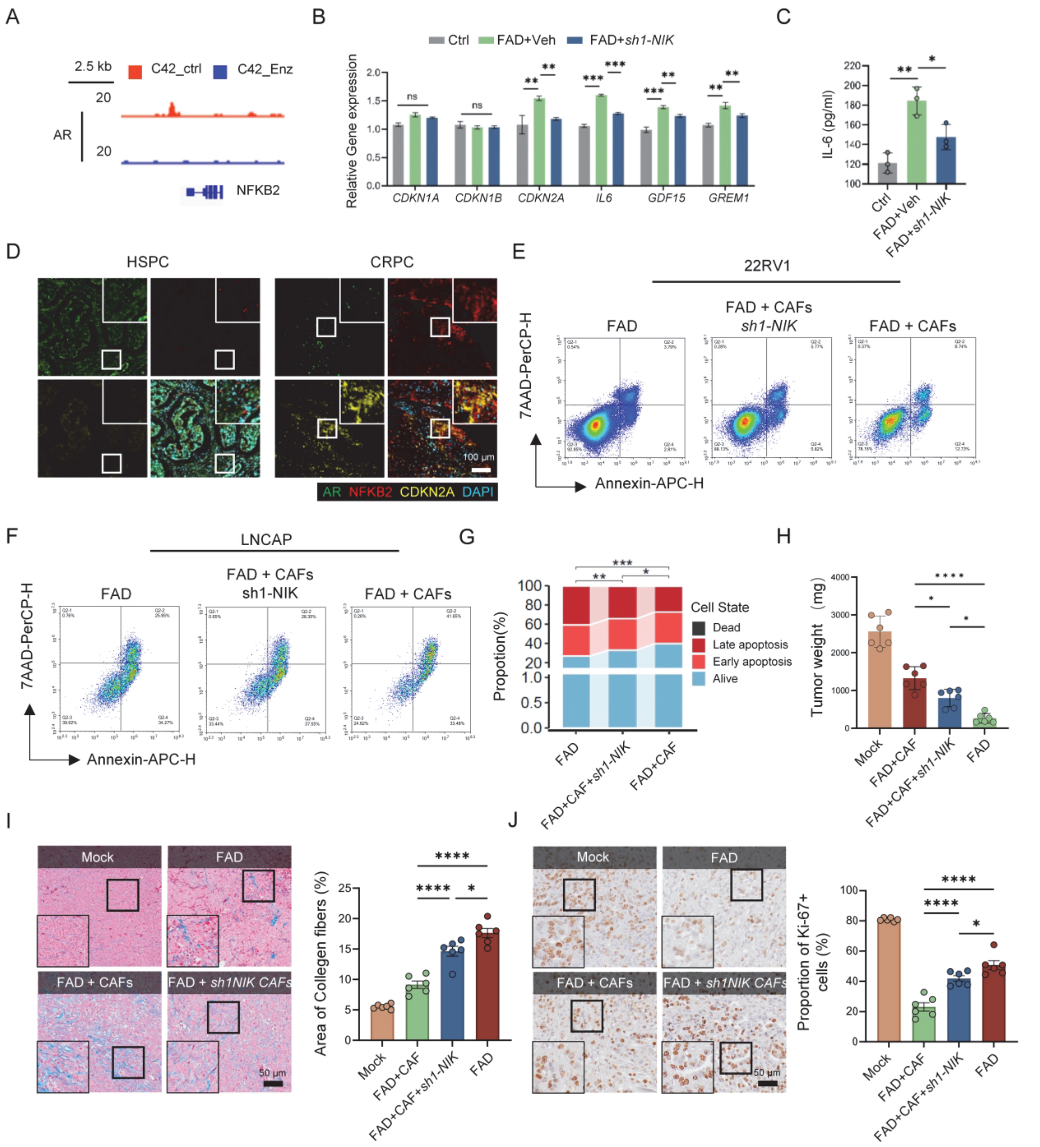

Figure S12. The AR-NFKB2 axis regulates CAF senescence and SASP (Related to Figure 6). **A,** ChIP-seq tracks showing AR binding peaks at the NFKB2 promoter region (C4-2 cells treated with Enzalutamide). **B,** RT-qPCR analysis of CDKN2A and SASP factors. **C,** ELISA for IL-6 secretion. **D,** Representative mIF images showing co-localization of NFKB2 and P16 in human CRPC tissues compared to HSPC. (scale bar = 100 μm). **E-G,** In vitro co-culture assays. Flow cytometry analysis of apoptosis in 22Rv1 (E) and LNCaP (F–G) cells co-cultured with control or shNIK-CAFs under FAD. **H-J,** In vivo xenografts related to Fig6.R. Tumor weights (H), representative Masson staining and quantification of collagen fiber area (scale bar = 50 μm) (I), and representative Ki-67 IHC staining and quantification of Ki-67^{+} proportion (scale bar = 50 μm). (J) in tumors co-injected with shNIK-CAFs compared to controls. Data are presented as mean ± SEM. Statistical significance was determined using one-way ANOVA or two-way ANOVA followed by post hoc tests, and unpaired t-test for two-group comparisons. *P < 0.05,**P < 0.01, ***P < 0.001.

Figure S13

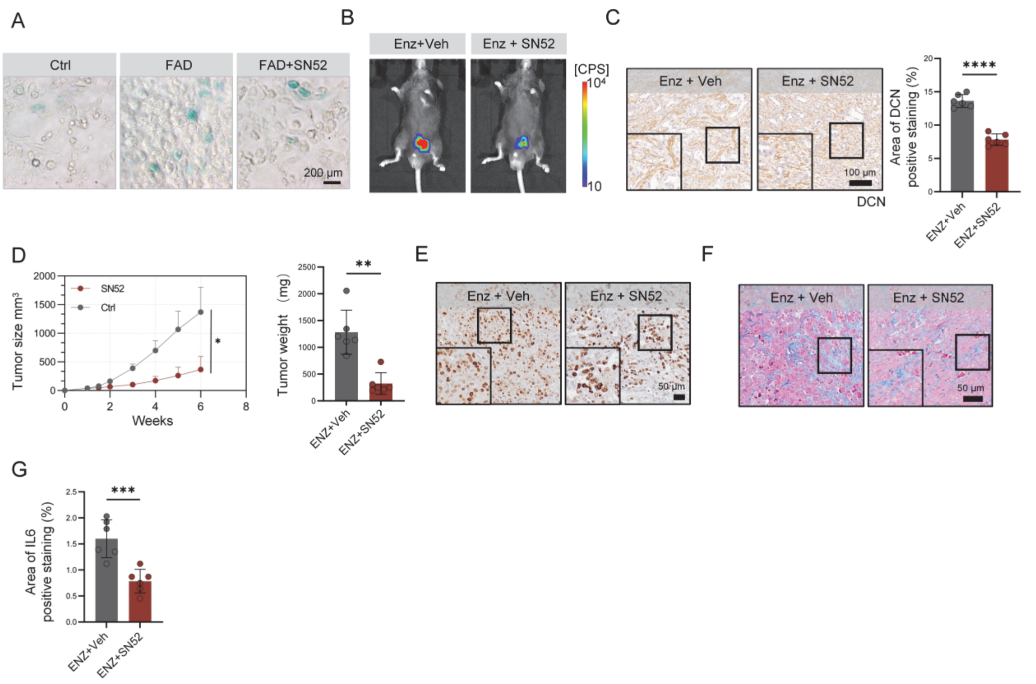

Figure S13. Pharmacological targeting of p52 with SN52 overcomes resistance (Related to Figure 7).

A, Representative SA-β-Gal staining. (scale bar = 200 μm). **B-C,** In vivo orthotopic model related to Fig7.K. Representative bioluminescence imaging (B) and representative DCN IHC staining and quantification of DCN^+ area (scale bar = 100 μm) (C) in mice treated with Enzalutamide (Enz) alone or in combination with SN52. **D-G,** In vivo subcutaneous humanized model related to Fig7.S. Tumor growth curves and final tumor weights (D). Representative Ki-67 IHC staining (scale bar = 50 μm) (E), and Masson staining and quantification of collagen fiber area (scale bar = 50 μm) (F). Data are presented as mean ± SEM. Statistical significance was determined using one-way ANOVA or two-way ANOVA followed by post hoc tests, and unpaired t-test for two-group comparisons. *$P < 0.05$,**$P < 0.01$, ***$P < 0.001$.
